# Caspase‐3/GSDME‐Mediated Trophoblast Pyroptosis and Reciprocal Macrophage Polarization Contribute to Inflammation in Early‐Onset Preeclampsia

**DOI:** 10.1002/advs.202516948

**Published:** 2026-01-20

**Authors:** Baoying Huang, Weinan Deng, Shilei Bi, Yifan Wang, Zhaowei Tu, Lijun Huang, Lili Du, Lizi Zhang, Zhoushan Feng, Wei Sun, Tengfei Liu, Julia Kzhyshkowska, Haibin Wang, Jingsi Chen, Dunjin Chen, Shuang Zhang

**Affiliations:** ^1^ Department of Obstetrics and Gynecology Guangdong Provincial Key Laboratory of Major Obstetric Diseases Guangdong Provincial Clinical Research Center For Obstetrics and Gynecology Guangdong‐Hong Kong‐Macao Greater Bay Area Higher Education Joint Laboratory of Maternal‐Fetal Medicine The Third Affiliated Hospital of Guangzhou Medical University Guangzhou China; ^2^ Institute of Transfusion Medicine and Immunology Mannheim Institute of Innate Immunosciences (MI3) Medical Faculty Mannheim Heidelberg University Mannheim Germany; ^3^ German Red Cross Blood Service Baden‐Württemberg‐Hessen Mannheim Germany; ^4^ Laboratory of Translational Cellular and Molecular Biomedicine National Research Tomsk State University Tomsk Russia; ^5^ Fujian Provincial Key Laboratory of Reproductive Health Research Department of Obstetrics and Gynecology The First Affiliated Hospital of Xiamen University School of Medicine Xiamen University Xiamen China

**Keywords:** preeclampsia, pyroptosis, trophoblast

## Abstract

Early‐onset preeclampsia (EOPE) is associated with excessive apoptosis and inflammation, but the mechanistic link between these processes remains enigma. Here, we report elevated circulating pro‐apoptotic proteins in EOPE patients at early pregnancy, along with concurrent CASP3 activation and GSDME cleavage in EOPE placentas. Using multiple trophoblast cell lines, we demonstrate that trophoblast cells, which highly express GSDME, undergo a shift from apoptosis to CASP3‐dependent pyroptosis, driving inflammation. Notably, pyroptotic trophoblasts further induce pro‐inflammatory macrophage polarization within placental villi organoids, establishing a feedback loop that amplifies both trophoblast pyroptosis and inflammatory responses in trophoblast organoids‐macrophage assembloids. In vivo, CASP3‐GSDME‐mediated trophoblast pyroptosis contributes to systemic inflammation in wild‐type pregnant mice but not in *Gsdme^−/−^* mice. Screening of EOPE prevention drugs reveals Vitamin D as a suppressor of GSDME activation and pyroptosis in trophoblast cells. Together, our findings establish CASP3–GSDME–mediated pyroptosis as a mechanistic link between apoptosis and inflammation in EOPE.

## Graphical abstract

In trophoblast cells, T/S (TNFα + SM164) activated caspase‐3, which cleaved GSDME to switch apoptosis to pyroptosis, causing cell swelling, membrane rupture, and release of LDH, HMGB1, IL‐1β and IL‐18. These cytokines induced pro‐inflammatory macrophage polarization, which in turn reinforced pyroptotic signaling in trophoblasts, amplifying systemic inflammation. Caspase‐3 inhibition or Vitamin D treatment attenuated this response.

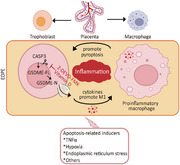

## 1 Introduction

Preeclampsia (PE) stands as a significant contributor to maternal and perinatal morbidity and mortality, afflicting 2 to 4 out of every 100 pregnancies worldwide [1, 2, 3, 4]. It manifests in two distinct forms delineated by gestational age at the time of diagnosis or delivery: early‐onset PE (EOPE, < 34 weeks) and late‐onset PE (LOPE, ≥ 34 weeks) [5]. Compared to late‐onset counterpart, early‐onset preeclampsia is considered to be associated with a greater incidence of the HELLP syndrome, abnormal uterine artery Doppler waveforms, atherosis, placenta lesions, small for gestational age, and fetal growth restriction [6, 7]. Presently, the sole remedy available for PE is the termination of pregnancy, a measure fraught with health risks for short and long‐term consequences of both the mother and the fetus [8, 9, 10, 11, 12, 13]. The pathogenesis of EOPE is primarily attributed to placental dysfunction, wherein the placenta releases pro‐inflammatory factors during the second stage, inciting a systemic inflammatory response that culminates in the clinical manifestations of PE [14]. Nonetheless, a comprehensive elucidation of the mechanisms underlying how placental abnormalities trigger the ensuing inflammatory cascade in PE remains elusive.

During placenta development, apoptosis plays a crucial role in cytotrophoblast fusion, syncytiotrophoblast turnover, and extravillous trophoblast invasion [15, 16]. Apoptosis is stimulated by factors like hypoxia and oxidative stress in placentation, triggering both extrinsic and intrinsic pathways that ultimately activate caspases and release apoptotic bodies into the maternal circulation [17]. Villous trophoblast apoptosis was observed to be higher in patients with PE compared to those with normal pregnancies [16, 18, 19]. However, the mechanisms underlying the activation of this excessive apoptosis and its contribution to the characteristics of PE remain largely unknown.

Pyroptosis, also known as inflammatory necrosis, is a caspase‐dependent program, showing DNA damage and chromatin condensation [20]. Unlike apoptosis, which does not trigger inflammation as apoptotic bodies has intact membrane and are phagocytosed [21], pyroptotic cells exhibit swelling and the appearance of bubble‐like protrusions on the cellular membrane before eventual rupture [22, 23]. Gasdermin proteins are key executors involved in pyroptosis, and their cleavage fragments form pores in the plasma membrane, thereby releasing pro‐inflammatory cytokines including IL‐1β and IL‐18, as well as HMGB1 and ATP [24]. There are six members of the human gasdermin family, including GSDM A‐D, GSDME (also known as DFNA5) and DFNB59 [25]. Specific gasdermin involved in pyroptosis depends on the context, the activation of caspase‐1 and caspase 11/4/5 cleave GSDMD and release the N‐terminal domain that can oligomerize to form pores in the cell membrane, while Caspase 3 inactivate GSDMD mediated pyroptosis by cleaving it at Asp87 site [26]. By contrast, GSDME is cleaved by active CASP3 at Asp270 to release a necrotic N‐GSDME fragment to induce pyroptosis directly [27], alternatively, N‐GSDME fragment in turn activates the NLRP3 inflammasome, leading to activation of the caspase 1/GSDMD cascade [28, 29]. GSDME mediated pyroptosis is increasingly reported to be involved in modulation of cancer progression and inflammatory diseases, while GSDME disruption skews pyroptotic death to apoptosis at sites of inflammation [30, 31, 32, 33]. Previous studies observed that, GSDME is most highly expressed in human placenta compared to other tissues [34, 35]. In a mouse model of Zika virus infection, GSDME activation induced pyroptosis of placental trophoblasts, resulting in placental defects and adverse fetal outcomes in infected pregnant mice [35]. Although these findings implicate GSDME in placental injury, it remains unclear whether GSDME mediates trophoblast pyroptosis under the pathological conditions of EOPE.

In this work, we demonstrate that GSDME, which is extensively expressed in trophoblast cells, switches CASP3 mediated apoptosis to pyroptosis, leading to a release of inflammatory cytokines. Remarkably, we elucidate a feedback loop between pyroptotic trophoblasts and pro‐inflammatory macrophages within EOPE placentas. These results highlight CASP3 as a predictive marker and position the CASP3‐GSDME pathway as a potential therapeutic target for preeclampsia prevention.

## 2 Results

### 2.1 High Relevance of CASP3 Activation and GSD+ME Cleavage in EOPE Placentas

**FIGURE 1:**
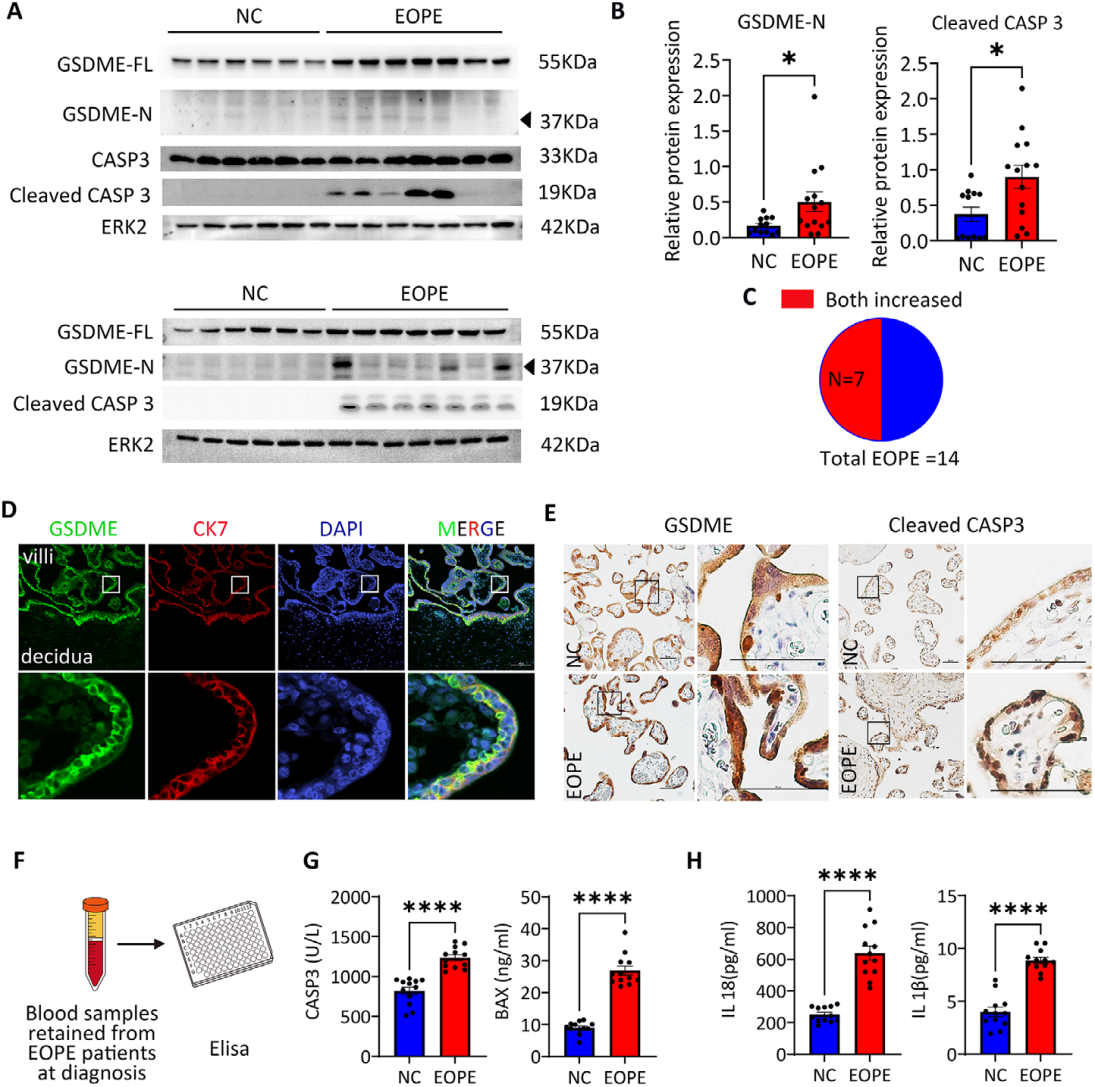
High Relevance of CASP3 Activation and GSDME Cleavage in a Subset of EOPE Placentas. (A) Immunoblots of pyroptosis‐related proteins (GSDME‐FL, GSDME‐N, CASP3 and cleaved CASP3) in lysate of placenta villi from NC and EOPE groups. ERK2 was used as a loading control. (B) Relative quantification of GSDME‐N, and cleaved CASP3 in the placentas by Western blot (controls(*n* = 12), EOPE(*n* = 14)). Error bars, mean ± SEM. The data were analyzed by Student's *t*‐test, ^*^ *p*<0.05. (C) Pie chart showing the proportion of EOPE patients exhibiting increased levels of both GSDME‐N and cleaved CASP3 in placental villous lysates. (D) Representative Immunofluorescence pictures of GSDME, CK7 and DAPI in the normal pregnant placenta villi and decidua at the third trimester. Scale bars, 100 µm. (E)Representative immunostaining pictures of GSDME‐N and cleaved CASP3 in the placenta of NC (*n* = 6) and EOPE (*n* = 6) group. Scale bars, 50 µm. (F) Schematic diagram of ELISA for detecting blood samples at diagnosis (24–25 weeks). (G) The CASP3 and BAX levels in plasma from patients with EOPE (*n* = 12) and controls (*n* = 12) were detected by ELISA kits. Error bars, mean ± SEM. The data were analyzed by Student's *t*‐test, ^***^ *p*<0.0001. (H) The IL‐1β and IL‐18 levels in plasma from patients with EOPE (*n* = 12) and controls (*n* = 12) were detected by ELISA kit. Error bars, mean ± SEM. The data were analyzed by Student's *t*‐test, ^***^ *p*<0.0001. NC, control; EOPE, Early‐onset preeclampsia.

Given that GSDME is highly expressed in trophoblasts and that its activator, caspase‐3 (CASP3), has been reported to be upregulated in EOPE placentas [28, 31, 34, 35], we investigated whether GSDME and CASP3 are associated in EOPE. To address this, we performed western blot analysis to examine CASP3, its active form (cleaved CASP3), and GSDME in placental samples from 14 EOPE patients and 12 gestational age‐matched preterm deliveries (see Figure S1A and Tables S1 and S2). We observed the activation of the apoptotic pathway in EOPE placentas, indicated by elevated levels of cleaved CASP3, in line with previous findings [36]. Notably, in 7 out of 14 EOPE placenta samples, GSDME‐N expression was significantly increased alongside CASP3 activation (Figure 1A–C), suggesting a possible link between CASP3 activation and GSDME cleavage in EOPE pathology. Immunofluorescence staining at maternal‐fetal interaction (both early and late pregnancy) revealed uniquely high cytoplasmic expression of GSDME in cytotrophoblast and syncytiotrophoblast cells, with relatively lower expression in maternal decidua tissue (Figure 1D; Figure S1B). Consistent with the immunoblot results, both GSDME and cleaved CASP3 were detected in EOPE placenta trophoblasts (Figure 1E). We also quantified apoptotic protein levels (BAX and CASP3) and GSDME mediated pyroptotic products including interleukin‐1β (IL‐1β) and IL‐18 [37, 38] by ELISA in blood samples obtained from EOPE patients at diagnosis and gestational age‐matched controls (Figure 1F; Table S2). Compared to the control group (*n* = 12), these patients (*n* = 12) exhibited significantly elevated levels of BAX, CASP3, IL‐1β and IL‐18 in plasma (Figure 1G,H; Table S2). These findings collectively indicating a strong association between CASP3 and GSDME in the inflammatory pathology of EOPE.

To further investigate whether CASP3 is associated with other inflammatory death pathways in EOPE placentas, we analyzed key proteins involved in pyroptosis (GSDME‐FL, GSDME‐N, GSDMD‐FL, GSDMD‐N, CASP1, CASP1 p20, CASP1 p10) and necroptosis (p‐RIPK3, RIPK3, p‐MLKL, MLKL) (Figure S1C,D). Although active GSDMD‐N and CASP1 (CASP1 p20) were detected in EOPE placentas, their levels did not differ significantly from the control group within the CASP3 active subset group (Figure S1C). Likewise, key necroptotic markers such as total RIPK3, phosphorylated RIPK3, total MLKL, and phosphorylated MLKL showed no significant changes between EOPE placentas and gestational age‐matched controls (Figure S1D). These findings suggest that CASP3 activation in EOPE placentas is not implicated in GSDMD‐mediated pyroptosis or necroptosis.

To investigate whether circulating apoptotic proteins are elevated in EOPE patients prior to diagnosis, we performed an OLINK proteomics in the blood samples of pregnant women between gestation weeks 12–16 who were subsequently diagnosed with EOPE (*n* = 5) and control groups (*n* = 4) (Figure S1E, Table S3). Among the proteins upregulated in the EOPE group, apoptotic markers such as BAX, CASP3, DECR1, and MAGED1, as well as the epithelial protein EPCAM, were of particular interest (Figure S1F). These findings suggest a strong association between excessive apoptosis and the early development of EOPE.

### 2.2 Excessive Apoptosis Induce GSDME‐Mediate Pyroptosis in Trophoblast Cells

**FIGURE 2:**
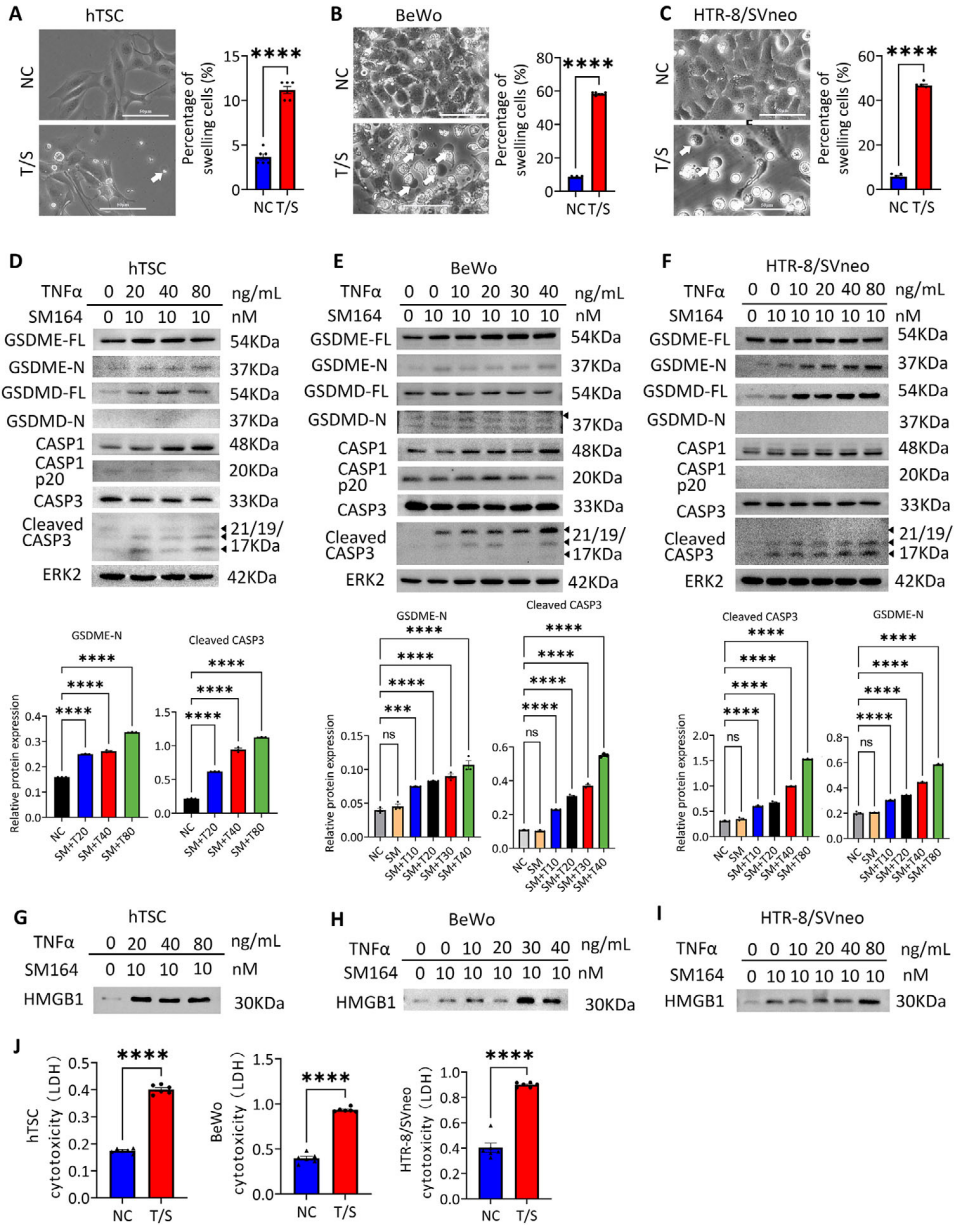
GSDME‐mediate pyroptosis was induced upon activation of apoptosis in trophoblast cells. (A–C) Phase‐contrast images of hTSC, BeWo cells and HTR‐8/SVneo cells treated with SM164 and TNFα (T/S here after) after 24 h. White arrows indicated the pyroptotic‐like cells. Scale bars, 50 µm. Percentage of swelling cells following T/S treatment. Data are representative of three independent experiments. Error bars, mean ± SEM. The data were analyzed by Student's *t*‐test, *n* = 3, duplicate each time, 3 fields of view were counted. ^****^ *p*<0.0001, ^***^ *p*<0.001. (D‐F) Immunoblots of GSDME‐FL, GSDME‐N, GSDMD‐FL, GSDMD‐N, CASP1, CASP1 p20, CASP3 and cleaved CASP3 in hTSC, BeWo cells and HTR‐8/SVneo cells treated with SM164 and different TNFα concentrations (0, 10, 20, 40, 80 ng/mL) after 24 h. ERK2 was used as a loading control. Relative quantification of GSDME‐N and Cleaved CASP3 in hTSC, BeWo cells and HTR‐8/SVneo cells treated with SM164 and different TNFα concentrations. Error bars, mean ± SEM. The data were analyzed with a one‐way ANOVA, *n* ≥ 3, ^****^ *p*<0.0001, ^***^ *p*<0.001, ^**^ *p*<0.01, ns, not significant. (G–I) Immunoblots of HMGB1 in the supernatant of hTSC, BeWo cells and HTR‐8/SVneo cells after treatment with T/S. (J) Cytotoxicity assay in hTSC, BeWo and HTR‐8/SVneo cell after T/S treatment based on the detection of released LDH. The experiments were performed in triplicates. Error bars, mean ± SEM. The data were analyzed by Student's *t*‐test, *n* ≥ 3, ^****^ *p*<0.0001. NC, control; T/S, SM164 and TNFα.

To confirm the above hypothesis, we utilized an apoptosis inducer, TNFα and SM164 (T/S hereafter), to trigger apoptosis in trophoblast cell lines including human trophoblast stem cells (hTSC), BeWo cells and HTR‐8/SVneo cells (Figure 2A) [39, 40]. Trophoblast cells underwent apoptosis under the condition of T/S, exhibiting cell shrinkage and fragmentation into apoptotic bodies (Figure 2A–C). We then conducted immunoblot on pyroptosis related proteins on trophoblast cell lines treated with different concentrations of T/S. Our results showed that cleaved CASP3 and GSDME‐N increased along with higher T/S concentration in all trophoblast cell lines including hTSC, BeWo cells and HTR‐8/SVneo cells (Figure 2D–F). In contrast, no significant changes were observed in CASP1 or GSDMD expression, suggesting that excessive apoptosis‐mediated pyroptosis in these cells is primarily driven through the CASP3‐GSDME axis. These findings were consistent with the results observed in EOPE patients (Figure S1C). High Mobility Group Box 1 (HMGB1), a Damage‐Associated Molecular Patterns (DAMP) molecule, is only released under conditions of cell lysis and serve as an indicator of pyroptosis [41, 42]. The supernatant was collected 24 h after T/S treatment and the protein level of HMGB1 was detected by western blot. As shown in Figure 2G–I, HMGB1 levels were significantly elevated at TNFα concentrations exceeding 20 ng/mL. Similarly, lactate dehydrogenase (LDH), an enzyme that can be detected in the process of pyroptosis when the cell plasma membrane breaks [43], was progressively elevated after T/S treatment in all trophoblast cells (Figure 2J). Notably, to further validate our findings, we conducted the aforementioned experiments using primary human trophoblasts (PHTs), and the results consistently aligned with those obtained from other cell lines (Figure S2). This reinforces the reliability of our data and conclusions.

In PHTs treated with brefeldin A (BFA, an ER stress inducer), cobalt chloride (CoCl_2_, a hypoxia mimic), or T/S (inducing excessive apoptosis), each representing different pathological conditions for preeclampsia, we observed pyroptosis‐like cellular morphology as shown in Figure S2A–F. Western blot analysis further demonstrated that all three induction regimens effectively induced the cleavage of GSDME‐N. Additionally, GSDMD cleavage was observed in the CoCl_2_ treated groups, consistent with a previous study [44, 45]. These findings suggest that pyroptosis can be activated through distinct molecular pathways under varying conditions.

To provide functional readouts of pyroptosis, we conducted additional experiments including time‐resolved PI uptake, TUNEL staining, LDH kinetics, and IF staining for GSDME membrane localization (Figure S3). Our results revealed that the proportion of PI positive cells (PI+) was time‐dependent increased upon T/S treatment (Figure S3A,B), suggesting escalating cell membrane compromise. Additionally, TUNEL staining showed a clear, time‐dependent increases in DNA fragmentation (Figure S3C,D), along with time‐dependent release of LDH in the corresponsive supernatant after T/S exposure (Figure S3E). GSDME proteins localization in cells treated without or with T/S for 24 h were visualized by immunofluorescence assays (Figure S3F), revealing diffusely distributed GSDME proteins in cytoplasm of BeWo cells prior T/S treatment, whereas membrane localized puncta after T/S treatment. Together, these observations affirm that T/S treatment induces pyroptotic cell death through GSDME activation.

### 2.3 Inhibition of GSDME or CASP3 Reduces Pyroptosis‐Like Phenotype

**FIGURE 3:**
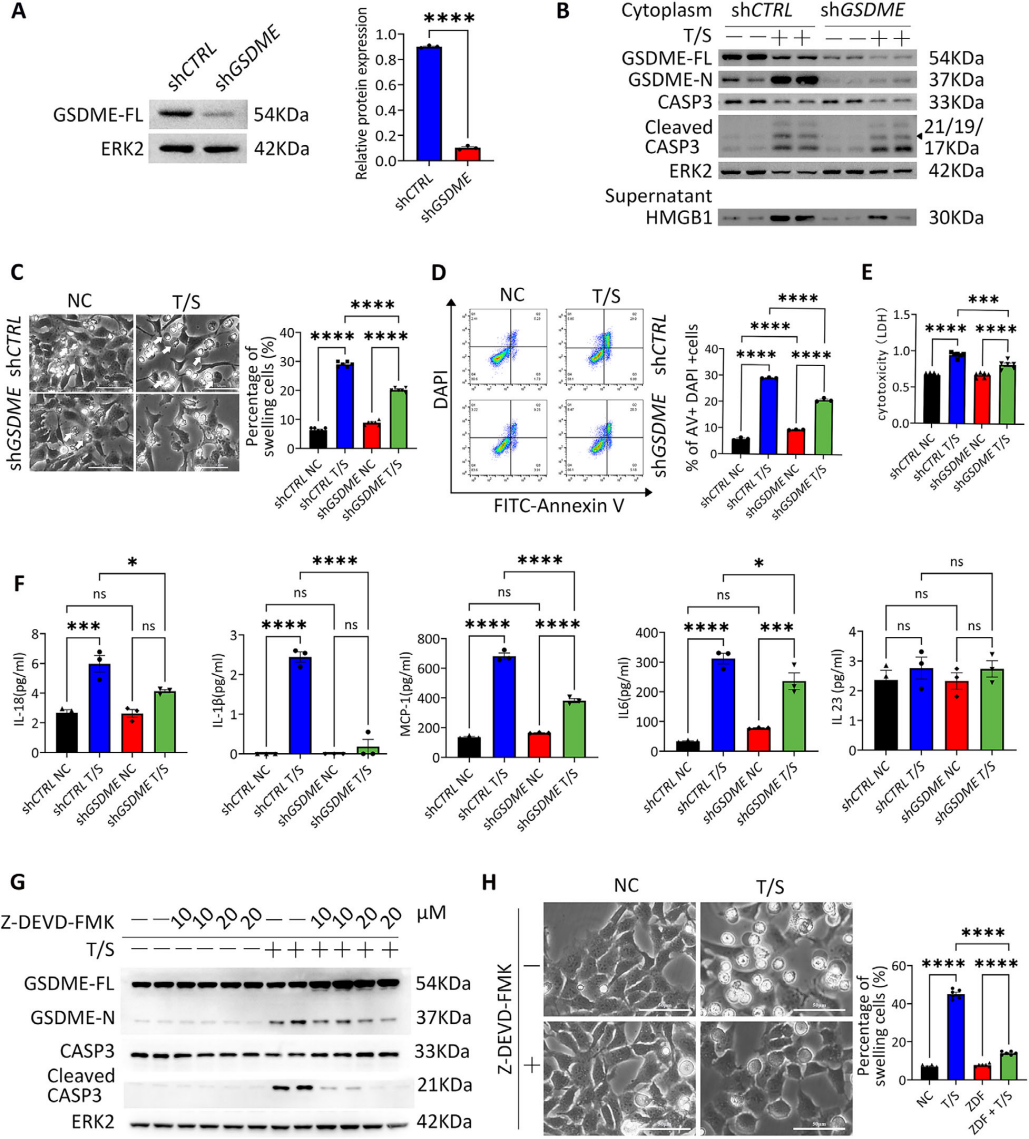
Inhibition of GSDME or CASP3 reduced pyroptosis‐like phenotype and inflammation in trophoblast cells. (A) Immunoblots of GSDME and its relative quantification in sh*CTRL* and sh*GSDME* in HTR‐8/SVneo cells. Error bars, mean ± SEM. The data were analyzed by Student's *t*‐test, *n* ≥ 3, ^****^ *p* < 0.0001. (B) Immunoblots of GSDME‐FL, GSDME‐N, CASP3, Cleaved CASP3 and HMGB1 in HTR‐8/SVneo cells (sh*CTRL* and sh*GSDME*) treated with T/S. ERK2 was used as a loading control. (C) Phase‐contrast images of sh*CTRL* and sh*GSDME* HTR‐8/SVneo cells at 24 h after T/S treatment. Arrows indicated the swelling cells. Scale bars, 50 µm. Percentages of swelling cells after T/S treatment. Error bars, mean ± SEM, *n* = 3, duplicate each time, 3 fields of view were counted. The data were analyzed with a one‐way ANOVA, ^****^ *p*<0.0001. (D) Flow cytometry of Annexin V and DAPI in sh*CTRL* and sh*GSDME* HTR‐8/SVneo cells treated with T/S. Percentages of Annexin V and DAPI double positive cells. Error bars, mean ± SEM, *n* ≥ 3. The data were analyzed with a one‐way ANOVA, ^****^ *p*<0.0001. (E) Comparison of LDH release of HTR‐8/SVneo cells (sh*CTRL* and sh*GSDME*) treated with T/S after 24 h. Error bars, mean ± SEM, n  = 3, duplicate each time. The data were analyzed with a one‐way ANOVA, ^****^ *p*<0.0001, ^***^ *p*<0.001. (F) Relative levels of IL‐1β, IL‐18, MCP‐1, IL‐6 and IL‐23 in the supernatant of cultured sh*CTRL* and sh*GSDME* HTR‐8/SVneo cells using the LEGENDplex inflammation panel. Error bars, mean ± SEM, *n* ≥ 3. The data were analyzed with a one‐way ANOVA, ^*^ *p*<0.05, ^***^ *p*<0.001, ^****^ *p*<0.0001, ns, not significant. (G) Immunoblots of GSDME‐FL, GSDME‐N, CASP3 and Cleaved CASP3 in HTR‐8/SVneo cells treated with T/S and Z‐DEVD‐FMK. ERK2 was used as a loading control. (H) Phase‐contrast images of HTR‐8/SVneo cells at 24 h after T/S or Z‐DEVD‐FMK treatment. Arrows indicated the swelling cells. Scale bars, 50 µm. Percentages of swelling cells after T/S treatment. Error bars, mean ± SEM, *n* = 3, duplicate each time, three representative fields per group were analyzed. The data were analyzed with a one‐way ANOVA, ^****^ *p*<0.0001, ^***^ *p*<0.001. NC, control; T/S, SM164 and TNFα.

To determine whether GSDME is the key director of pyroptosis in trophoblast cells, we used shRNA to knockdown the expression of GSDME in trophoblast cells. The expression of GSDME was efficiently decreased with sh*GSDME* infection in HTR‐8/SVneo cells and BeWo cells as detected by western blot (Figure 3A; Figure S4A). Immunoblot analysis further demonstrated significantly reduced GSDME expression and N‐terminal cleavage in the sh*GSDME* group compared to sh*CTRL* following T/S treatment, despite robust CASP3 activation in both groups (Figure 3B). Consistent with reduced GSDME cleavage, the increase in HMGB1 by T/S treatment in sh*GSDME* group was not as robust as that in sh*CTRL* cells (Figure 3B). Interestingly, we observed the swelling cells in sh*GSDME* trophoblast cells upon T/S treatment were not as robust as the sh*control* (sh*CTRL*) group (Figure 3C; Figure S4B,C). Additionally, pyroptotic cell death was identified as Annexin V+/ DAPI+ cells that simultaneously exhibited pyroptotic characteristics were reduced, primarily due to a decrease in pyroptosis (Figure 3D). The release of LDH was also decreased with GSDME knockdown in both HTR‐8/SVneo cells and BeWo cells (Figure 3E; Figure S4D).

It had been reported that GSDMD and GSDME perpetuate inflammation by mediating the release of cytokines such as IL‐1β and IL‐18 [37, 38]. As demonstrated in Figure 3F, T/S treatment significantly enhanced the secretion of both IL‐1β and IL‐18. Notably, GSDME knockdown partially attenuated this inflammatory response, as evidenced by reduced expression levels of these cytokines following T/S stimulation.

We next introduced the CASP3 inhibitor, Z‐DEVD‐FMK [46, 47], to evaluate whether GSDME‐mediated pyroptosis is CASP3 dependent. As shown in Figure 3G, immunoblot analysis confirmed that both the N‐terminal fragment of GSDME and cleaved CASP3 were significantly decreased in the Z‐DEVD‐FMK treated group compared to the control group after T/S treatment. Consistently, we observed a significant reduction in pyroptotic‐like cells in Z‐DEVD‐FMK treated trophoblast cells following T/S treatment compared to the control group. (Figure 3H). These findings suggest that reducing CASP3 activation or GSDME cleavage can alleviate pyroptosis.

### 2.4 Pyroptotic Trophoblasts Promote Pro‐Inflammatory Macrophage Polarization Within Placenta Villi Organoids

**FIGURE 4:**
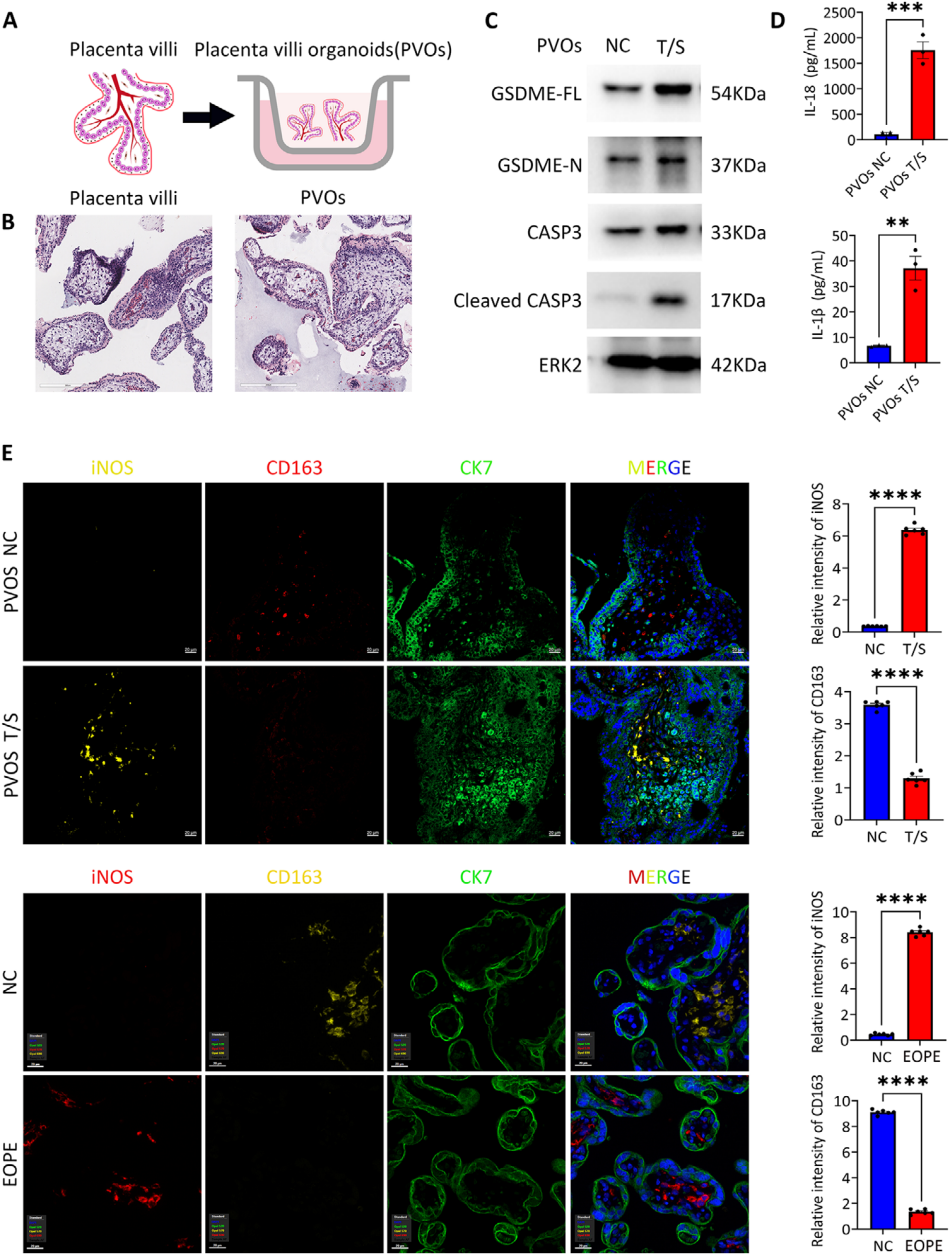
Pyroptotic trophoblasts drive pro‐inflammatory macrophage polarization within placenta villi organoids. (A) Schematic diagram of placenta villi organoids (PVOs) construction. (B) Haematoxylin and eosin (H&E) staining of placenta villi and PVOs. (C) Immunoblots of pyroptosis‐related proteins (GSDME‐FL, GSDME‐N, CASP3 and Cleaved CASP3) in lysate of PVOs treated T/S after 24 h. ERK2 was used as a loading control. (D) IL‐18 and IL‐1β level in the supernatant of PVOs treated T/S after 24 h using the ELISA kit. Error bars, mean ± SEM. The data were analyzed by Student's *t*‐test, *n* ≥ 3, ^***^ *p*<0.001, ^**^ *p*<0.01. (E) Representative Immunofluorescence staining images of iNOS, CD163, CK7 and DAPI of PVOs treated T/S after 24 h. Scale bars, 20 µm. Representative Immunofluorescence staining images of iNOS, CD163, CK7 and DAPI in the NC and EOPE placentas. Scale bars, 20 µm. The quantification of relative level of iNOS and CD163 in the PVOs treated T/S after 24 h. Error bars, mean ± SEM. The data were analyzed by Student's *t*‐test, *n* = 3, duplicate each time, three representative fields per slide were analyzed, ^****^ *p*<0.0001. The quantification of relative level of iNOS and CD163 in the NC (*n* = 6) and EOPE (*n* = 6) placentas. Error bars, mean ± SEM. The data were analyzed by Student's *t*‐test, *n* = 6, three representative fields per slide were analyzed, ^****^ *p*<0.0001. NC, control; T/S, TNFα and SM164.

Fetal‐origin Hofbauer macrophages have recently been identified as key mediators of inflammation, particularly in EOPE, but not in LOPE [48, 49]. To explore the impact of excessive apoptosis on placenta microenvironment during early pregnancy, we isolated placenta villi and cultured them as organoids (PVOs), which well retained both trophoblast cells and immune cells, following the protocol described in our previous publication [50] (Figure 4A). Morphologically, the PVOs exhibited a close resemblance to native placental villi after one week of in vitro culture (Figure 4B). The PVOs treated with T/S showed increased CASP3 activation and GSDME cleavage (Figure 4C), as well as elevated levels of inflammatory cytokines such as IL‐1β and IL‐18 in the supernatant (Figure 4D), in line with the observations in other trophoblast cells. Interestingly, we observed that macrophages co‐cultured with T/S pre‐treated organoid, displayed an inflammatory phenotype, showing decreased CD163, the classical M2 macrophage marker, and increased iNOS, a M1 macrophage marker, reflecting the imbalance of M1 iNOS+/CD163+ observed in PVOs of EOPE patients (Figure 4E).

### 2.5 Pyroptotic Trophoblasts and Pro‐Inflammatory Macrophages Form a Feedback Loop That Accelerates Inflammation

**FIGURE 5:**
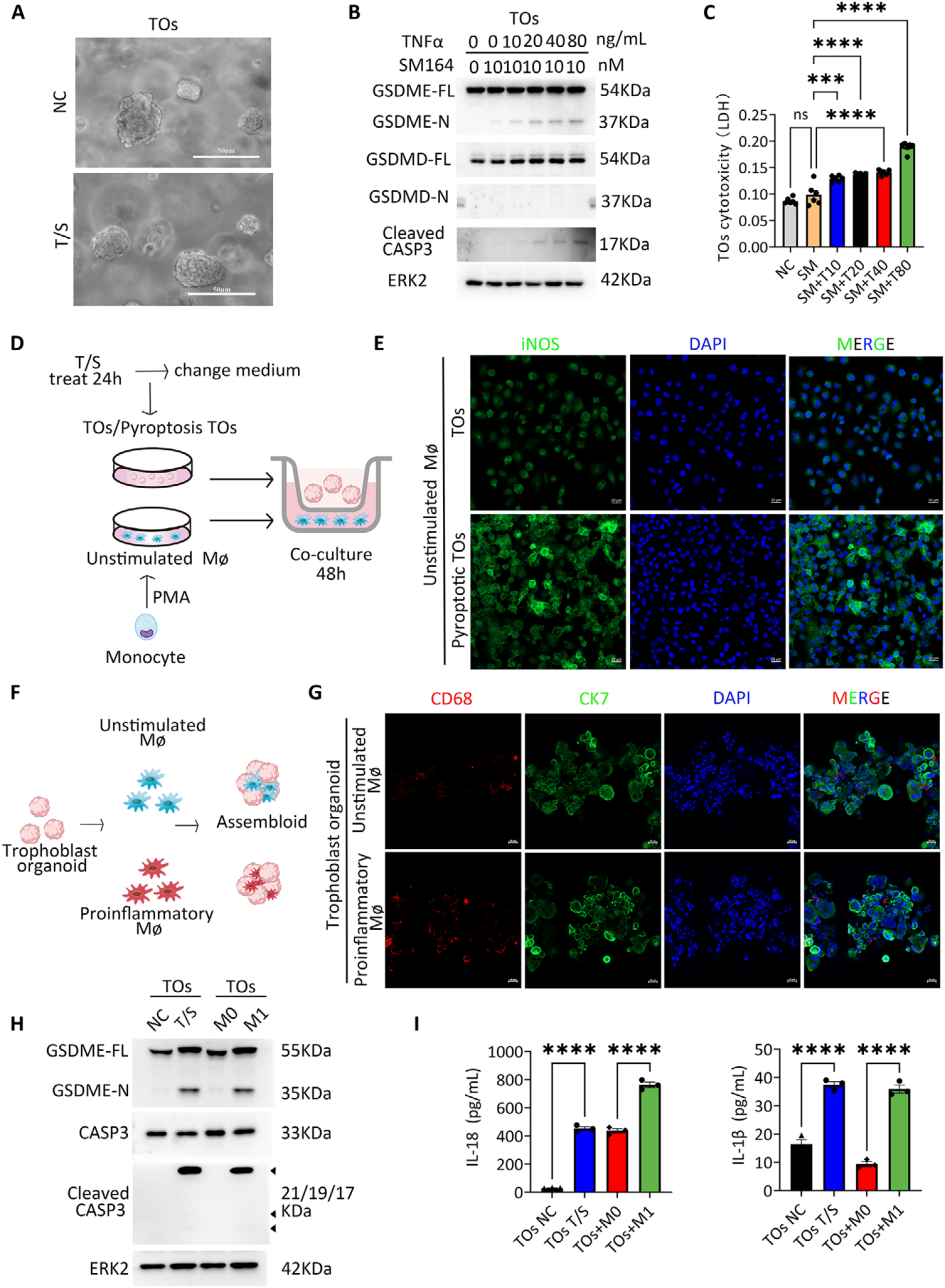
Pyroptotic trophoblasts and pro‐inflammatory macrophages influence each other, promoting the inflammatory response. (A)Phase‐contrast images of TOs treated with T/S after 24 h. Scale bars, 50 µm. (B) Immunoblots of GSDME‐FL, GSDME‐N, GSDMD‐FL, GSDMD‐N and Cleaved CASP3 in TOs treated with SM164 and different TNFα concentrations (0, 10, 20, 40, 80 ng/mL) after 24 h. ERK2 was used as a loading control. (C) Cytotoxicity assay in TOs cell after T/S treatment based on the detection of released LDH. Error bars, mean ± SEM, *n* = 3, duplicate each time. The data were analyzed with a one‐way ANOVA, ^****^ *p*<0.0001, ^**^ *p*<0.01, ^*^ *p*<0.05. (D) Schematic diagram of TOs and macrophage (Mφ) co‐culture model construction. (E) Representative Immunofluorescence staining images of iNOS after co‐culture of macrophages in the basal state with pyroptotic and non‐pyroptotic TOs. Scale bars, 20 µm. (F) Schematic diagram of TOs and Mφ assembloid construction. (G) Composite z stack confocal images of TOs and Mφ assembloid at day 3 after reaggregation containing unstimulated or proinflammatory macrophages stained with the antibodies against CD68, CK7 and DAPI. Scale bars, 20 µm. (H) Immunoblots of pyroptosis‐related proteins (GSDME‐FL, GSDME‐N, CASP3 and Cleaved CASP3) in lysate of TOs treated T/S after 24 h and TOs and Mφ assembloid containing unstimulated or proinflammatory macrophages. ERK2 was used as a loading control. (I) IL‐18 and IL‐1β level in the culture supernatant of TOs treated T/S after 24 h and TOs and Mφ assembloid containing unstimulated or proinflammatory macrophages using the ELISA kit. Error bars, mean ± SEM, *n* ≥ 3. The data were analyzed with a one‐way ANOVA. ^****^ *p*<0.0001. NC, control; T/S, TNFα and SM164.

To examine the potential association between macrophage polarization and trophoblast pyroptosis, we employed human trophoblast organoids (TOs) derived from placenta villi. Using this model system, we systematically evaluated the response of TOs to varying concentrations of T/S treatment. As expected, cleaved CASP3 and GSDME‐N levels increased with higher T/S concentrations in TOs (Figure 5A,B), consistent with observations in other trophoblast lines. Furthermore, LDH levels also elevated in parallel with T/S concentrations, further validating TOs as a suitable model for studying the effect of pyroptotic trophoblast cells on macrophage polarization (Figure 5C).

The interaction between macrophages and trophoblast cells was then evaluated through establishing a co‐culture model of macrophage and TOs (Figure 5D). THP‐1 cells were subjected to a 48 h treatment regimen involving PMA, thereby facilitating the acquisition of unstimulated macrophage. TOs were treated with T/S for 24 h, after which the medium was replaced, and co‐cultures of TOs and unstimulated macrophage were maintained for 48 h using transwell‐polycarbonate inserts. Immunofluorescence staining revealed in the T/S‐treated TOs, the expression of pro‐inflammatory macrophages marker iNOS was significantly increased (Figure 5D,E), indicating that pyroptotic trophoblasts promote pro‐inflammatory polarization of macrophages.

To investigate the influence of pro‐inflammatory and unstimulated macrophages on trophoblast cells, we constructed an assembloid model combining macrophage and TOs (Figure 5F,G). Initially, THP‐1 cells were subjected to a 48 h treatment regimen involving PMA alone as well as PMA in conjunction with IFNγ, thereby facilitating the acquisition of unstimulated macrophage and pro‐inflammatory macrophages. Pro‐inflammatory macrophage induced increased expression of GSDME‐N and cleaved CASP3 in TOs, suggesting their role in driving pyroptosis in trophoblasts (Figure 5H). Furthermore, we measured IL‐18 and IL‐1β levels in the culture supernatants of assembloid and T/S‐treated TOs after 24 h using ELISA. Both the T/S‐treated TOs and pro‐inflammatory macrophage assembloid showed significantly increased levels of IL18 and IL1β (Figure 5I), which were consistent with findings in EOPE placenta (Figure 1H) and PVOs (Figure 4D). These results suggest that pro‐inflammatory macrophage polarization drive trophoblasts pyroptosis. Taken together, our findings highlight the crosstalk between pyroptotic trophoblasts and pro‐inflammatory macrophage within the placenta villi, creating a feedback loop that exacerbates trophoblast pyroptosis and amplifies inflammatory cascades.

To determine whether inhibition of GSDME pore formation disrupts the trophoblast‐ macrophage feedback loop in PVOs, we treated PVOs with dimethyl fumarate (DMF), a covalent succinating agent that modifies critical cysteine residues in gasdermins and thereby blocks their oligomerization and pore formation, preventing gasdermin‑mediated pyroptosis (Figure S5A) [51, 52]. The efficacy of DMF was first validated in HTR8/SVneo cells by immunoblotting analysis, showing inhibited GSDME cleavage induced by T/S treatment (Figure S5B). Consistently, DMF administration significantly alleviated cell swelling and bubbling along with reduced LDH release caused by T/S induction (Figure S5C–E). Moreover, DMF treatment led to a significant reduction in GSDME‐mediated inflammation‐related cytokines such as IL‐1β, IL‐18 (Figure S5F). In PVOs, DMF treatment abolished the pro‐inflammatory shift in macrophages and reduced LDH release (Figure S5G–I). This anti‐inflammatory effect was further supported by decreased levels of IL‐1β and IL‐18 (Figure S5J) and a reduction in iNOS+ macrophages compared to T/S‐treated controls. These findings indicate that GSDME pore formation blockade limits trophoblast pyroptosis and disrupts the downstream pro‐inflammatory macrophage response in our PVO model.

### 2.6 CASP3‐GSDME Mediated Placenta Pyroptosis Contributes to Systemic Inflammation In Vivo

**FIGURE 6:**
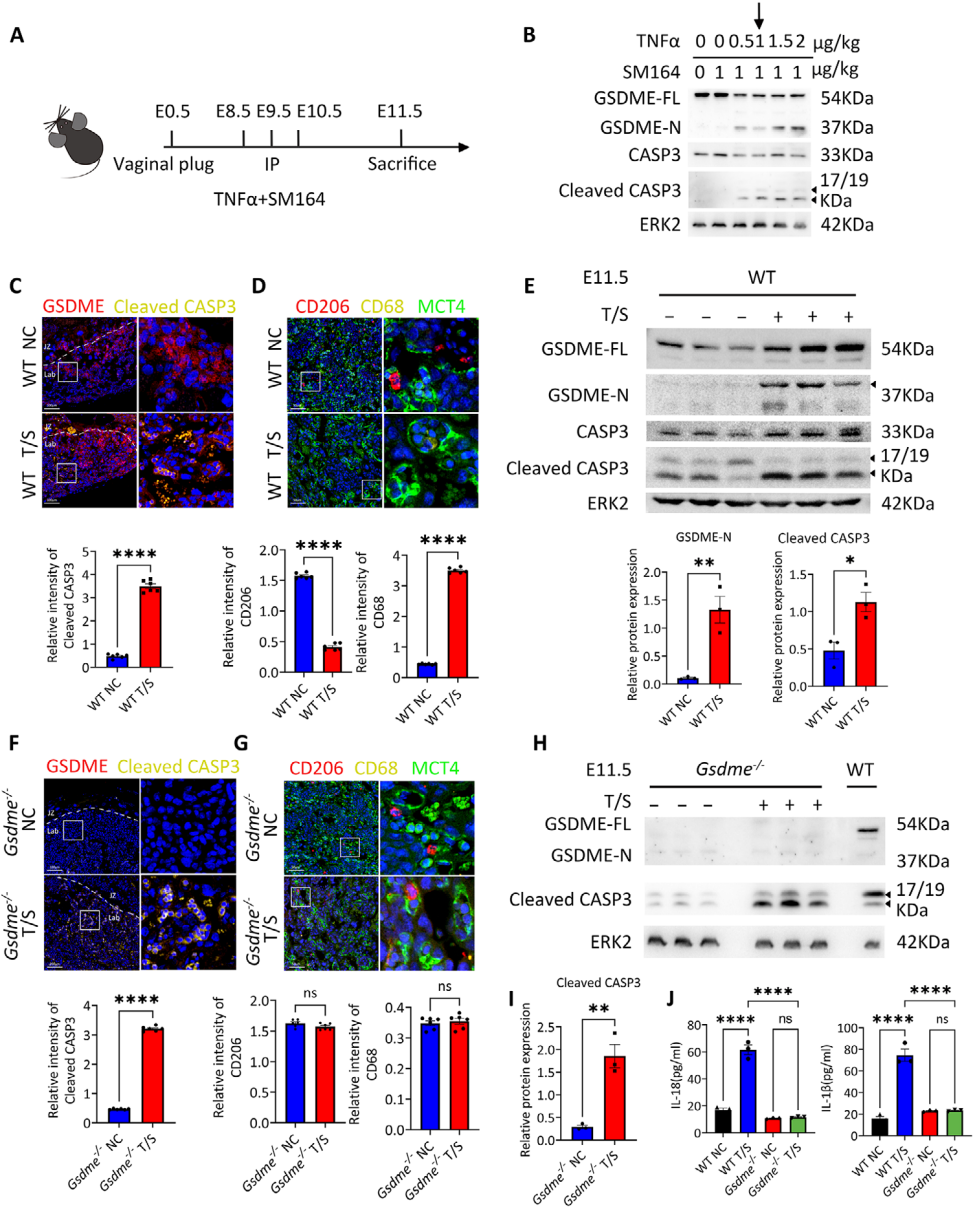
Caspase‐3‐GSDME‐mediated placental pyroptosis plays a role in promoting systemic inflammation in vivo. (A) Schematic diagram of the T/S treatment protocol in mice. (B) Immunoblots of GSDME‐FL, GSDME‐N, CASP3 and Cleaved CASP3 in placentas of WT mice treated with TNFα plus SM164. ERK2 was used as a loading control. (C) Representative immunofluorescence staining of GSDME and cleaved CASP3 in placental tissue from T/S‐treated mice. The quantification of relative level of Cleaved CASP3 in placental tissue from T/S‐treated mice. Error bars, mean ± SEM. The data were analyzed by Student's *t*‐test, *n* = 3, duplicate each time, three representative fields per slide were analyzed, ^****^ *p*<0.0001. Scale bars, 100 µm. (D) Representative immunofluorescence staining of MCT4, CD206, and CD68 in placental tissue from T/S‐treated mice. Scale bars, 50 µm. The quantification of relative level of CD206 and CD68 in placental tissue from T/S‐treated mice. Error bars, mean ± SEM. The data were analyzed by Student's *t*‐test, *n* = 3, duplicate each time, three representative fields per slide were analyzed, ^****^ *p*<0.0001. (E) Immunoblots of GSDME‐FL, GSDME‐N, CASP3 and Cleaved CASP3 in placental tissue from T/S‐treated mice. ERK2 as the loading control. Relative quantification of GSDME‐N and cleaved CASP3 in placental tissue from T/S‐treated mice, as determined by Western blot. Error bars, mean ± SEM. The data were analyzed by Student's *t*‐test, *n* ≥ 3, ^**^ *p*<0.01, ^*^ *p*<0.05. (F) Representative Immunofluorescence staining images of GSDME and Cleaved CASP3 and CD206, CD68 in placental tissue from T/S‐treated *Gsdme^−/−^* mice. Quantification of immunofluorescence signals for Cleaved CASP3 in placental tissue from T/S‐treated *Gsdme^−/−^* mice. Error bars, mean ± SEM. The data were analyzed by Student's *t*‐test, *n* = 3, duplicate each time, three representative fields per slide were analyzed, ^****^ *p*<0.0001. Scale bars, 100 µm. (G) Representative Immunofluorescence staining images of MCT4, CD206 and CD68 in placental tissue from T/S‐treated *Gsdme^−/−^* mice. Scale bars, 50 µm. Quantification of immunofluorescence signals for CD206 and CD68 in placental tissue from T/S‐treated *Gsdme^−/−^* mice. Error bars, mean ± SEM. The data were analyzed by Student's *t*‐test, *n* = 3, duplicate each time, three representative fields per slide were analyzed, ns, not significant. (H) Immunoblots of GSDME‐FL, GSDME‐N and Cleaved CASP3 in placental tissue from T/S‐treated *Gsdme^−/−^* mice. ERK2 as the loading control. (I) Relative quantification of GSDME‐N, Cleaved CASP3 in placental tissue from T/S‐treated *Gsdme^−/−^* mice. Error bars, mean ± SEM. The data were analyzed by Student's *t*‐test, *n* ≥ 3, ^**^ *p*<0.01. (J) IL‐18 and IL‐1β level in the serum of T/S‐treated WT and T/S‐treated *Gsdme^−/−^* mice using the ELISA kits. Error bars, mean ± SEM. The data were analyzed with a one‐way ANOVA, *n* ≥ 3, ^****^ *p*<0.0001, ns, not significant. NC, control; T/S, TNFα and SM164.

To explore whether local placental inflammation induced by GSDME‐mediated trophoblast pyroptosis and its crosstalk with pro‐inflammatory macrophages contributes to systemic inflammation in vivo, we simulated the heightened apoptosis observed in EOPE, by administering T/S to pregnant mice at embryonic days 8.5 (E8.5), E9.5 and E10.5. Age‐matched control mice received injections of 0.9% NaCl. Placenta and fetuses were collected at E11.5 to evaluate the impact of T/S treatment (Figure 6A). Initially, we tested various doses of TNFα ranging from 0.5 to 2 µg/kg and analyzed placental samples via immunoblotting. As shown in Figure 6B, a dose‐dependent increase in CASP3 activation and GSDME cleavage were observed, in line with our in vitro findings. We selected TNFα at 1 µg/kg for subsequent investigations, as this concentration initiated the observed phenotype. T/S treatment significantly enhanced CASP3 activation and GSDME cleavage in mouse placenta. To assess fetal macrophage status, we co‐stained macrophage markers CD68/CD206 with MCT4, a marker specifically expressed in the SynT‐II layer of the labyrinth zone that directly interfaces with the fetal blood spaces [53, 54]. Consistent with our in vitro experiments, pro‐inflammatory macrophage CD68 was upregulated and anti‐inflammatory macrophage marker CD206 was downregulated (Figure 6C–E).

To ascertain whether local GSDME activation in the placenta contributes to systemic inflammation, we utilized a *Gsdme* knockout mouse model (*Gsdme^−/−^*). Following the same injection protocol as in wild‐type mice (Figure 6A), immunofluorescence stain and western blot analysis of placenta revealed a complete absence of GSDME expression in *Gsdme^−/−^* placentas, confirming effective GSDME knockout (Figure 6F–H; Figure S6). Furthermore, CASP3 activation was still upregulated following T/S treatment. GSDME knockout abolished the pro‐inflammatory shift in macrophages (Figure 6F–I).

Notably, we observed significantly elevated levels of the inflammatory cytokines IL‐1β and IL‐18 in the serum of T/S‐treated mice compared to control mice, while this increase was attenuated in *Gsdme^−/−^* mice (Figure 6J), suggesting that GSDME‐induced pyroptosis in mouse placenta contributes to systemic inflammation in vivo.

### 2.7 Screening of EOPE Prevention Drugs Reveals Vitamin D Has a Role in Suppressing GSDME Activation and Trophoblast Cells Pyroptosis

**FIGURE 7:**
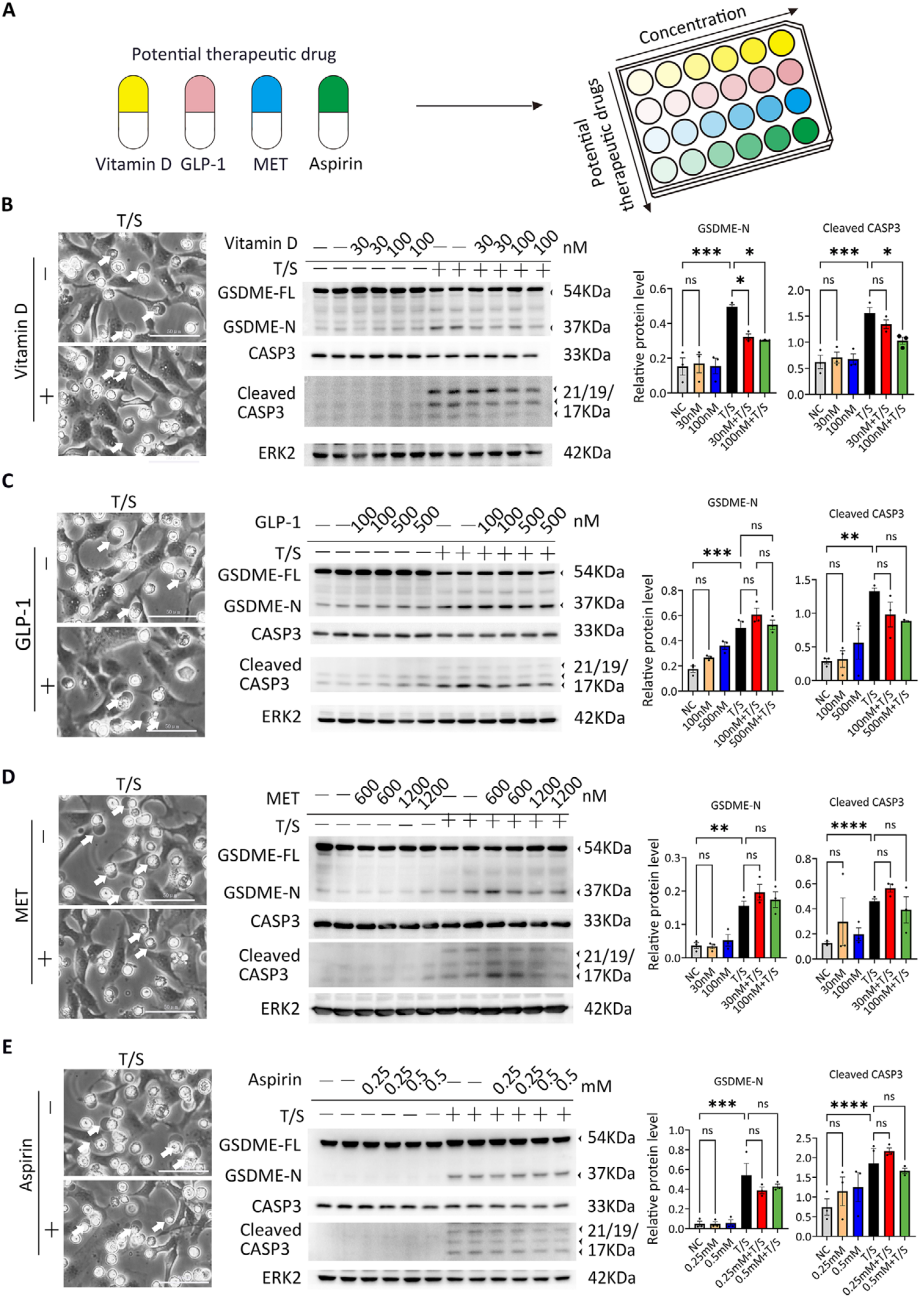
Screening the Effect of EOPE Prevention Drugs on Inhibiting Caspase‐3‐GSDME‐Mediated Pyroptosis. (A) Schematic diagram of drug screen for Vitamin D, GLP‐1, MET and aspirin in HTR‐8/SVneo cells. (B) Phase‐contrast images, immunoblots of GSDME‐FL, GSDME‐N, CASP3 and Cleaved CASP3 of HTR‐8/SVneo cells treated with Vitamin D and T/S after 24 h. Arrows, the swelling cells. Scale bars, 50 µm. (C) Phase‐contrast images, immunoblots of GSDME‐FL, GSDME‐N, CASP3 and Cleaved CASP3 of HTR‐8/SVneo cells treated with GLP‐1 and T/S after 24 h. Arrows, the swelling cells. Scale bars, 50 µm. (D) Phase‐contrast images, immunoblots of GSDME‐FL, GSDME‐N, CASP3 and cleaved CASP3 of HTR‐8/SVneo cells treated with MET and T/S after 24 h. Arrows, the swelling cells. Scale bars, 50 µm. (E) Phase‐contrast images, immunoblots of GSDME‐FL, GSDME‐N and Cleaved CASP3 of HTR‐8/SVneo cells treated with Aspirin and T/S after 24 h. Arrows, the swelling cells. Scale bars, 50 µm. Relative quantification of GSDME‐N and Cleaved CASP3 in the HTR‐8/SVneo cells treated with Vitamin D, GLP‐1, MET and aspirin by western blot. (B–E). Error bars, mean ± SEM. The data were analyzed with a one‐way ANOVA, *n* ≥ 3, ^****^ *p*<0.0001, ^***^ *p*<0.001, ^**^ *p*<0.01, ^*^ *p*<0.05, ns, not significant. NC, control; T/S, TNFα and SM164; GLP‐1, Glucagon‐like peptide‐1; MET, Metformin.

Several medications are currently used, such as low‐dose aspirin [55], or have been proposed as potential candidates, including vitamin D and metformin [56, 57], for the prevention of preeclampsia. In this study, we investigated whether these drugs, together with a GLP‐1 antagonist—previously reported to exert pleiotropic anti‐inflammatory effects with a favorable safety profile [58]—could suppress GSDME activation (Figure 7A). As shown in Figure 7, trophoblast cells treated with vitamin D exhibited reduced GSDME cleavage upon T/S induction. In contrast, treatments with GLP‐1, aspirin and metformin (MET) did not result in a notable decrease in either pyroptotic cell death or GSDME cleavage (Figure 7B–E). Collectively, these findings highlight vitamin D as a promising candidate for preventing preeclampsia in patient subset with high CASP3–GSDME activity, although targeted, high‐quality randomized controlled trials are warranted to validate its clinical efficacy.

## 3 Discussion

We demonstrated that GSDME in trophoblast cells switched CASP3 mediated apoptosis to pyroptosis, leading to cell swelling and inflammation by releasing LDH, HMGB1 and inflammatory cytokines IL‐18 and IL‐1β. GSDME knockdown mitigated these adverse effects, and targeting activation of CASP3 by Z‐DEVD‐FMK reduced inflammation in trophoblast cell lines. These findings provide genetic evidence supporting the role of trophoblasts pyroptosis in mediating systemic inflammation of EOPE patients and the potential of targeting the CASP3‐GSDME pathway as a new prevention strategy for preeclampsia. While our current sample size is statistically sufficient to validate these mechanistic insights, we acknowledge that a larger cohort will be essential to further assess clinical translatability and patient variability. Future studies with expanded patient samples are warranted to strengthen the generalizability and feasibility of this approach.

Our comparative analysis of placental samples from EOPE patients and age‐matched pregnancies revealed a higher expression of the activated form of GSDME (N‐GSDME) in EOPE placenta. Subsequent investigations on multiple trophoblast models indicated that CASP3 cleaves GSDME, converting trophoblast cell apoptosis to pyroptosis under conditions of consecutive apoptosis induction. This mechanism parallels observations in tumor cell death induced by chemotherapeutic agents [28, 29]. Notably, a previous study reported active Caspase‐1, increased GSDMD cleavage, IL‐1β, and IL‐18 in EOPE placenta and primary human trophoblasts under hypoxia [44]. In our study, we similarly observed GSDMD cleavage during hypoxia induced by CoCl_2_ treatment, but not under T/S. In contrast, GSDME cleavage was evident under apoptotic conditions, hypoxia, and BFA treatment, coinciding with CASP3 activation, underscoring its critical role in mediating pyroptosis across diverse pathological stresses. Taken together, although GSDMD may be involved under certain stress conditions, our data primarily support a CASP3–GSDME–mediated pyroptosis pathway rather than a universal CASP1/NLRP3–GSDMD axis driving pyroptosis downstream of apoptosis.

Besides, we observed in this study that the serum IL‐1β and IL‐18 levels track the pyroptotic status of the mice placenta, but in *Gsdme^−/−^* mice, this linkage is attenuated, suggesting that GSDME‐mediated pyroptosis in placental tissue contributes to systemic inflammation. Although systemic inflammation can also arise from immune cells or other maternal cells affected by systemic *Gsdme* deficiency, our immunofluorescence analysis of the maternal–fetal interface reveals that GSDME is scarcely expressed in the maternal decidua yet highly expressed in trophoblasts. This pattern implies a prominent role for trophoblast pyroptosis in modulating systemic inflammatory signals. In general, these findings support that placental GSDME‐induced pyroptosis contributes substantially to systemic inflammation, though it may not be the sole driver in this process. To further substantiate this mechanism, future studies employing direct placental targeting strategies—such as placenta‐specific delivery of LPN55–*siGsdme*—are warranted to precisely delineate the contribution of GSDME‐mediated pyroptosis to systemic inflammatory responses.

Fetal‐origin Hofbauer macrophages have recently been identified as key mediators of inflammation, particularly in EOPE, but not in LOPE [48, 49]. To replicate the conditions of excessive trophoblast cells apoptosis observed in EOPE, we treated these PVOs isolated from 6 to 8 weeks pregnancies with an apoptosis inducer. Notably, this treatment led to an increase in M1 macrophages, resembling the inflammatory macrophage profile observed in placental villi during late pregnancy. These results hightlight excessive apoptosis as a primary trigger for the initiation of EOPE, aligning with previous studies [19]. Furthermore, co‐culture experiments of macrophages with trophoblast spheroids (TOs) or assembloids comprising both cell types provided robust evidence that pyroptotic trophoblasts and pro‐inflammatory macrophages establish a self‐reinforcing inflammatory cascade within EOPE placentas. In our study, we utilized THP‐1‐derived macrophages as an in vitro model to simulate trophoblast‐induced responses due to their robustness and reproducibility in mechanistic studies. Nevertheless, further validation of our findings in primary first‐trimester fetal macrophages to strengthen translational relevance will be valuable in the future.

The TNFα‐infusion in pregnant animals, such as mice [59], rats [60, 61], and baboons [62, 63] has been well‐documented to generate features indicative of PE, like hypertension, renal impairment, and proteinuria, although it does not result in fetal growth retardation. Our primary focus was on placental trophoblast defects, representing an early stage in the development of early EOPE. Additionally, the early detection of CASP3 as a potential biomarker for EOPE, which can be identified in patients as early as 12–16 weeks, preceding the typical clinical diagnosis typically occurring after 24 weeks. To investigate this further, we administered TNFα and SM164 to mice during the early placental development stage (E8.5–E10.5) within a short observational window, rather than subjecting the pregnant mice to long‐term challenges. Apart from pyroptosis, necroptosis represents another pro‐inflammatory form of cell death, releasing intracellular damage‐associated molecular patterns (DAMPs) that promote inflammation [64]. Necroptotic morphology has been observed in placentas of mice exhibiting preeclampsia‐like symptoms [65]. In late preeclampsia, trophoblast cells undergo necroptotic cell death induced by ceramide [66]. Key necroptosis effector molecules, including RIPK1 and SIRT2, have been detected in placental tissues of both normal pregnancies and those with PE [67]. However, a recent study reported no evidence of necroptosis in placental tissue from EOPE patients [44], suggesting distinct inflammatory mechanisms between EOPE and LOPE. Consistent with this finding, we observed no significant alterations in necrosis, as evidenced by the minimal detection of p‐RIPK3 and p‐MLKL in EOPE placenta.

**FIGURE 8:**
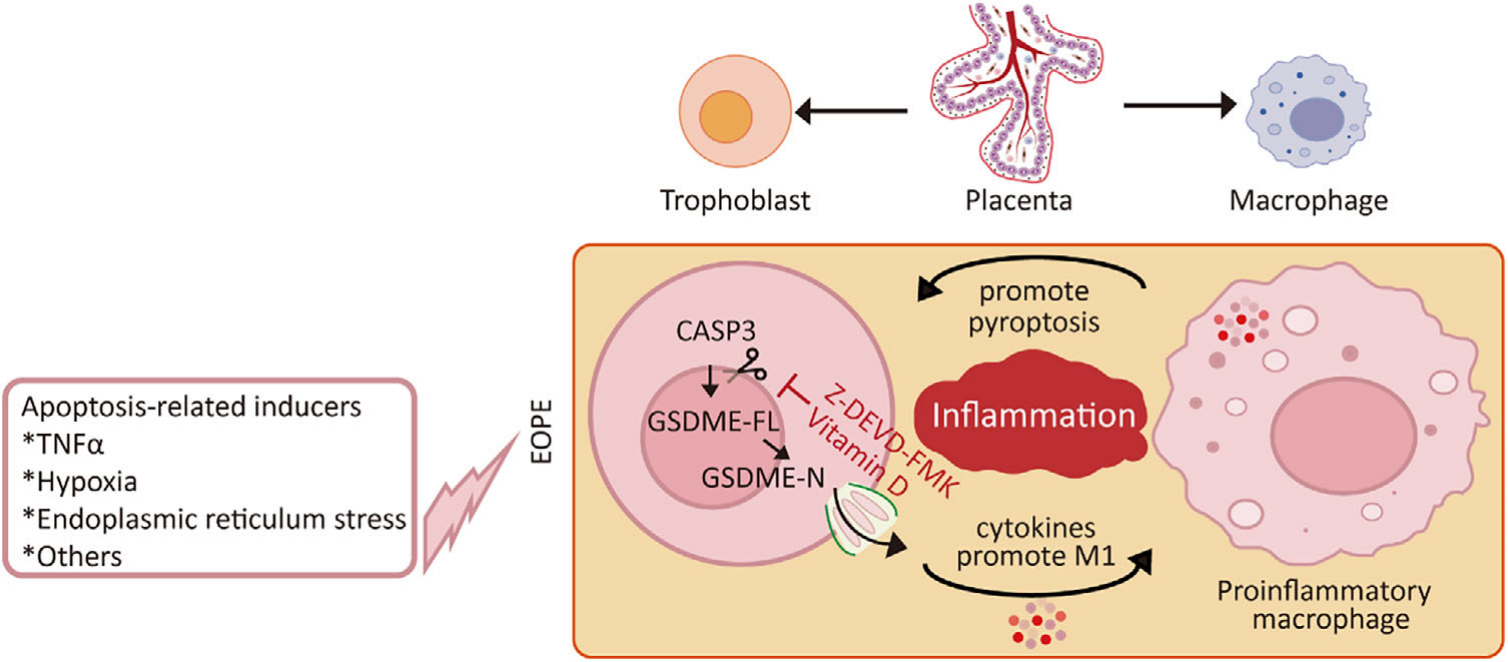
Pyroptotic trophoblasts interact with and activate pro‑inflammatory macrophages, contributing to systemic inflammation. In trophoblast cells, stimulation with the apoptotic inducer T/S (TNFα + SM164) activated caspase‐3, which cleaved GSDME and triggered a switch from classical apoptosis to pyroptosis. This transition was characterized by cell swelling, membrane rupture, and the release of LDH, HMGB1, and proinflammatory cytokines IL‐1β and IL‐18. The secreted cytokines promoted the polarization of pro‐inflammatory macrophages, which in turn reinforced pyroptotic signaling in trophoblasts, amplifying systemic inflammation. Pharmacological inhibition of caspase‐3 with Z‐DEVD‐FMK or treatment with Vitamin D significantly attenuated pyroptosis associated inflammatory responses. CASP3, the activated (cleaved) form of caspase‐3.

In conclusion, we show that GSDME‑high trophoblasts transition from apoptosis to CASP3‑dependent pyroptosis, triggering inflammatory signaling. These pyroptotic trophoblasts further drive pro‑inflammatory macrophage polarization within placental villi organoids, establishing a feed‑forward loop that amplifies both trophoblast pyroptosis and inflammation in trophoblast–macrophage assembloids (Figure 8). Although current systematic reviews and meta‑analyses report inconsistent evidence regarding vitamin D supplementation in preeclampsia prevention [57], our findings identify vitamin D as a suppressor of GSDME activation. Future large‑scale randomized controlled trials—particularly those enriched with excessive apoptosis (such as CASP3^hi^) patient cohorts—may provide a more accurate evaluation of vitamin D's therapeutic potential in EOPE.

## 4 Materials and Methods

### 4.1 Human Placentas Tissues Collection

Tissue samples used for this study were obtained with written informed consent from participants. The study was approved by the Medical Ethics Committee of the Third Affiliated Hospital of Guangzhou Medical University, Medical Research (No. 2018002). Placentas tissues at 28–34 weeks of gestation from EOPE patients or age‐matched healthy pregnancies were collected from women who underwent caesarean deliveries from January 2017 to October 2022 at the Third Affiliated Hospital of Guangzhou Medical University, Guangdong, China. The diagnostic criteria for EOPE were established based on guidelines outlined by the International Society for the Study of Hypertension in Pregnancy [68]. EOPE is characterized by the onset of hypertension occurring after 20 weeks of gestation, presenting with a systolic blood pressure of ≥140 mmHg and/or diastolic blood pressure of ≥90 mmHg, accompanied by proteinuria at 0.3 g/day. Alternatively, EOPE may manifest without proteinuria, but with the presence of one or more of the following symptoms: thrombocytopenia, liver function impairment, renal function impairment, pulmonary edema, neurological abnormalities, intrauterine growth restriction, or uteroplacental insufficiency. Normal pregnant controls do not exhibit pregnancy complications such as gestational or chronic hypertension, pregestational or gestational diabetes mellitus, or intrahepatic cholestasis of pregnancy. Collected tissues were snap‐frozen in dry ice at the time of surgery and stored at −80°C or liquid nitrogen. Part of the tissue was fixed in 4% PFA for histopathological analysis. Detailed information of patients is listed in Tables S1–S3.

### 4.2 Blood Sample Collection

Blood samples used for the retrospective proteomic study of early‐onset preeclampsia (EOPE) were obtained from the biobank of the Third Affiliated Hospital of Guangzhou Medical University. The samples were collected during routine clinical practice at 12–16 weeks of gestation from consenting patients who were subsequently diagnosed with EOPE at the same hospital between 2019 and 2020 (Detailed information is listed in Table S1). Plasma was prepared from whole blood collected into tubes with EDTA by centrifugation at 3000 rpm for 15 mins at room temperature, after which the plasma was aliquoted and frozen for long‐term storage at −80°C until analysis.

### 4.3 Blood Sample Proteome Profiling

The protein levels of all samples were measured in plasma using the Olink Explore 1536, which uses antibody‐binding capabilities to detect the level a combination of 4 separate Olink Explore 384 panels, including Cardiometabolic panel, Inflammation panel, Neurology panel and Oncology panel. Data is presented as NPX (Normalized Protein eXpression) values, which is relative protein quantification unit on log2 scale.

### 4.4 Animals

The animals were housed in the animal care facility of Guangzhou Medical University according to the guidelines for the care and use of laboratory animals (Ethics Number: 2022–618). All experimental procedures were approved by the Animal Welfare Committee of Research Organization of Guangzhou Medical University. Two‐month‐old C57BL/6 wild‐type mice were purchased from Guangdong Medical Laboratory Animal Center and used in the experiments. *Gsdme^−/−^* mice were obtained from Prof. Feng Shao laboratory at National Institute of Biological Sciences, Beijing, the mice were constructed as previously reported [28]. The wild‐type and *Gsdme^−/−^* female mice were mated with wild‐type male and *Gsdme^−/−^* male mice respectively, and the pregnant mice were injected with TNFα (1 µg/kg) and SM164 (1 µg/kg) or with an equal volume of 0.9% NaCl at embryonic day 8.5 (E8.5), E9.5 and E10.5 of pregnancy. The mice were sacrificed at E11.5 and the placentas, fetues and serum were collected for indicated experiments. The genotyping primers for *Gsdme^−/−^* mice are as follows: F1: 5′−tCCATTACTGTGGCTAAAGAGGGGC −3′; R1: 5′−CTTCCTAAACTCCTGCGGAAGACAG −3′ R2: 5′−GGCCTCTAGCCCAGCATGC −3′.

### 4.5 Primary Trophoblast, Trophoblast Lines, Trophoblast Stem Cell and Trophoblast Organoids Culture

The isolation of PHTs from full‐term placentas was performed as described previously [69]. Placentas were collected immediately after cesarean section from uncomplicated singleton pregnancies at term. Exclusion criteria included fetal anomalies, maternal pathologies, or placental abnormalities. Tissue was transported on ice and processed within 1 h. Amniotic membranes were removed, and placenta was diced into 2 × 2 cm pieces. Tissue was washed with 0.9% NaCl (4–8 L) until the wash solution became clear. Vessels, chorionic and decidual plates, and blood clots were meticulously dissected to minimize contamination. 72 g of minced tissue was incubated in 300 mL pre‐warmed (37°C) 1× HBSS (Thermo, Cat. 14185052) containing 720 U dispase II (Sigma, Cat. D4693) for 1 h under agitation. DNase I (100 U/mL, Worthington, Cat. LS006344) was added for an additional 15 min to reduce clumping. The digest was sequentially filtered through 200, 100, and 40 µm sieves. The filtrate was centrifuged (500 × g, 20 min), and the pellet was resuspended in wash medium (DMEM/F12 + 1% Penicillin‐Streptomycin). A discontinuous Percoll gradient (70%–5%, 3 mL layers) was overlaid with 6 mL of resuspended cells and centrifuged (1200 × g, 20 min, no brake). The cytotrophoblast layer (density 1.048–1.062 g/mL, fractions 45%–35%) was aspirated, washed, and treated with 0.05% trypsin (1 min, 37°C, Gibco, Cat. 25300062) followed by DNase I (100 U/mL). Cells were filtered (40 µm), counted (trypan blue exclusion), and viability >90% was confirmed. Cells were plated at 4.0 × 10^5 cells/cm^2 in DMEM/F12 medium (Corning, Cat. 10‐092‐CV) containing 10% fetal bovine serum (FBS, Lifeman, Cat.FBS001) and 1% Penicillin‐Streptomycin (Gibco, Cat. 15140122) under 21% O_2_/5% CO_2_ at 37°C. Medium was changed daily. Mycoplasma testing (YEASEN, 40601ES20) was performed on PHTs, and no contamination was detected.

Human trophoblast stem cells (hTSC) were isolated from placenta during the first trimester (6–8 weeks of gestation) as previously reported [70]. hTSCs were cultured in hTSC medium supplemented with 0.1 mm 2‐mercaptoethanol (Thermo Fisher Scientific, Cat. 21985023), 0.2% FBS (Corning, Cat. 26219002), 0.5% Penicillin‐Streptomycin (Gibico, Cat. 15140122), 0.3% BSA (Sigma, Cat. A9418), 1% ITS‐X supplement (Wako, Cat. 094–06761), 1.5 mg/mL L‐ascorbic acid (Wako, Cat. 013–12061), 50 ng/mL EGF (Wako, Cat. 053–07871), 2 mm CHIR99021 (Wako, Cat. 034–23103), 0.5 mm A83‐01 (Wako, Cat. 035–24113), 1 mm SB431542 (Wako, Cat. 031–24291), 0.8 mm VPA (Wako, Cat. 227–01071) and 5 mm Y27632 (Wako, Cat. 036–24023). Mycoplasma testing (YEASEN, 40601ES20) was performed on hTSC, and no contamination was detected.

The methodology for establishing trophoblast organoids (TOs) was adapted from our previously published article [71]. The hTSC were harvested and re‐suspended in ice‐cold basic trophoblast organoid medium (bTOM) containing advanced DMEM/F12(Gibco, Cat.12634010) supplemented with 10 mm HEPES (Gibco, Cat. 15630080), B27 (Gibco, Cat. 17504044), N2 (Gibco, Cat.17502048) and 2 mm L‐glutamine (Life Technologies, Cat. 25030–081). The cells were then centrifuged for 3 min at 1000 rpm following which the cells were re‐suspended in ice‐cold advanced trophoblast organoid medium (aTOM) which is bTOM supplemented with 100 ng/mL R‐spondin (PeproTech, Cat.HZ‐1328), 1 µm A8301 (Wako, Cat. 035–24113), 100 ng/mL recombinant human epidermal growth factor (rhEGF, Peprotech, Cat. AF‐100‐15), 50 ng/mL recombinant human hepatocyte growth factor (rhHGF, PeproTech, Cat. 100–39), 2.5 µm prostaglandin E2 (Sigma, Cat. P0409), 3 µm CHIR99021 (Wako, Cat. 034–23103) and 100 ng/mL Noggin (PeproTech, Cat. 120‐10c). Growth factor reduced matrigel (Corning, Cat. 356231) was added to the aTOM cell suspension to reach a final concentration of 60%. 50 µL of the cell solution containing 2.5 × 10^4^ cells was plated in the center of a 24‐well plate. The solution rests as a dome‐shaped droplet in the center of the well. The plates are then turned upside down and kept at 37°C for 15–30 min to ensure proper spreading of the cells in the solidifying matrigel domes. Finally, the plates are returned to their upward position and the domes are overlaid with 500 µL of room temperature aTOM medium. The organoids are allowed to form for 10 days with fresh media being changed every 2 days. Mycoplasma testing (YEASEN, 40601ES20) was performed on TOs, and no contamination was detected.

The methodology for establishing placenta villi organoids (PVOs) was adapted from our previously published article [50]. Collagen solutions were mixed as follows: Cellmatrix I‐A (Wako, Cat. 637–00653), Advanced DMEM/F12 (Gibco, Cat. 12634010), 20 mm HEPES (Thermo Fisher Scientific, Cat. 15630080) at a ratio of 8:1:1. The mixture was kept on ice to prevent gel formation until use. The formation of air bubbles should be avoided during mixing. To prepare the culture dish, Millicell culture plate inserts (Millipore, Cat. R1KB36634) with permeable membrane bottoms were placed in a 60 mm tissue culture dish. To create the bottom layer, 1 mL of the prepared reconstituted collagen solution was added to the inserts under sterile conditions. The culture dish with the inserts was incubated in an incubator at 37°C for 30 min. Villi slices or villi were washed with 10 mL basal medium and resuspended ∼0.1 mg in 1 mL reconstituted collagen solution. The mixtures were layered on top of the pre‐solidified bottom layer to form the double dish air‐liquid culture system as described. 2 mL placenta villi organoid culture medium was added into the culture dish on the outer layer. The culture medium was changed twice a week. The basal medium consisted of Advanced DMEM/F12 (Gibco, Cat. 12634010), 10 mm HEPES (Thermo Fisher Scientific, Cat. 15630080), 1X GlutaMAX (Life Technologies, Cat. 2268102), and 1% penicillin/streptomycin (Gibco, Cat. 15140‐122). The placenta villi organoid culture medium consisted of the basal medium supplemented with 10 mm nicotinamide (Sigma, Cat. N0636), 1X B‐27 (Invitrogen, Cat. 12587010), 1 mm N‐acetylcysteine (Sigma, Cat. A9165), 10 µm SB431542 (Wako, Cat. 031–24291), 0.5 mm A83‐01 (Wako, Cat. 039–24111), 100 ng/mL EGF (Wako, Cat. 053–07871), 100 ng/mL FGF2 (Peprotech, Cat. 450‐33), 50 ng/mL HGF (Peprotech, Cat. 100–39), 2 µm Y‐27632 (Wako, Cat. 030–24021), 2.5 µm PGE2 (Sigma, Cat. P0409), and 50% (v/v) WRN‐CM. WRN‐conditioned medium was obtained from a commercially available ATCC CRL‐3276 (ATCC) cell line.

Mycoplasma testing (YEASEN, 40601ES20) was performed on PVOs, and no contamination was detected.

HTR8/SVneo (ATCC Cat# CRL‐3271, RRID: CVCL_7162) and BeWo (ATCC Cat# CCL‐98, RRID: CVCL_0044) cell lines were obtained from the American Type Culture Collection (ATCC, Manassas, VA, USA) authenticated using STR profiling by ATCC and tested to be free from mycoplasma contamination (YEASEN, 40601ES20). HTR8/SVneo cells were grown in 10 cm cell culture dish with 1640 medium (Corning, Cat. 10‐040‐CV) containing 10% fetal bovine serum (FBS, Lifeman, Cat.FBS001) and 1% Penicillin‐Streptomycin (Gibco, Cat. 15140122). BeWo cells were cultured in 10 cm cell culture dish with DMEM/F12 medium (Corning, Cat. 10‐092‐CV) containing 10% fetal bovine serum (FBS, Lifeman, Cat.FBS001) and 1% Penicillin‐Streptomycin (Gibco, Cat. 15140122). All cells were cultured at 37°C in 5% CO_2_ and the culture medium was replaced every two days. Static bright field cell images were captured using the Leica microscope (Leica, Cat. DMIL LED).

### 4.6 THP‐1 Cells Culture and Differentiation

THP‐1 cell lines (RRID: CVCL_0006) were obtained from the American Type Culture Collection (ATCC, Manassas, VA, USA) authenticated using STR profiling by ATCC and tested to be free from mycoplasma contamination (YEASEN, 40601ES20). THP‐1 cells were grown in 10 cm cell culture dish with 1640 medium (Corning, Cat. 10‐040‐CV) containing 10% fetal bovine serum (FBS, Lifeman, Cat.FBS001), 0.05 mm 2‐mercaptoethanol (Thermo Fisher Scientific, Cat. 21985023) and 1% Penicillin‐Streptomycin (Gibco, Cat. 15140122). THP‐1 cells (1× 10^6^/well) were seeded into 6‐well plates in the presence of 100 ng/mL phorbol 12‐myristate 13‐acetate (PMA, MCE, Cat.HY‐18739) and Interleukin 4 (IL 4, PeproTech, Cat. 200–04)or Interferon gamma(IFNγ, PeproTech, Cat. 300‐02) for 48 h. Then, the medium was removed and THP‐1 cells were cultured with fresh RPMI 1640 medium for an additional 24 h. For the indicated experiments, the PMA‐treated THP‐1 cells were co‐cultured with TSO for up to 48 h.

### 4.7 Macrophages and Trophoblast Cells Co‐Culture System

The interaction between macrophages and trophoblast cells was then evaluated through establishing a co‐culture model of macrophage and TOs. THP‐1 cells were subjected to a 48 h treatment regimen involving PMA, thereby facilitating the acquisition of unstimulated macrophage. TOs were treated with T/S for 24 h, after which the medium was replaced, and co‐cultures of TOs and unstimulated macrophage were maintained for 48 h using transwell‐polycarbonate inserts.

### 4.8 Construction of TO‐ Macrophage Assembloids

The TO‐macrophage assembloids were constructed with TSO and THP‐1 cells‐ induced macrophages. Briefly, TOs were released with Cultrex Organoid Harvesting Solution (R&D, Cat. 3700‐100‐01) at Day 10–12 and macrophages were dissociated with Accutase after day 3 of the differentiation procedure.

The TOs and macrophages were reaggregated with approximately 95%–98% TOs and approximately 2%–5% macrophages in advanced trophoblast organoid medium (aTOM) supplemented with 10% fetal bovine serum (FBS, Lifeman, Cat.FBS001) using low‐attach U plates. 48 h later, the cells self‐assembled into organoids.

### 4.9 Plasmids Construction and shRNA Knockdown

The shRNA of GSDME Were Designed by Genetic Perturbation Platform from BROAD institute and Primers (F: 5’‐ CCGGGCTTCTAAGTCTGGTGACAAACTCGAGTTTGTCACCAGACTTAGAAGCTTTTTG‐3’; R:5’‐AATTCAAAAAGCTTCTAAGTCTGGTGACAAACTCGAGTTTGTCACCAGACTTAGAAGC‐3’) were synthesized by Shanghai General Biological Engineering Co, LTD. The *shRNAs* were cloned into the pLKO.1‐Puro lentiviral vector. To generate lentiviral vectors, HEK293FT cells were cotransfected with lentiviral vectors, psPAX2, and pMD2.G at a ratio of 10:7.5:2.5 using jetPRIME Transfection Reagent (polyplus, Cat.114‐15), following the manufacturer's instructions. Media were replaced 8 h post‐transfection, and viral supernatants were collected 72 h later after passing through a 0.45 µm filter. The collected supernatants were aliquoted and stored at −80°C. To achieve GSDME knockdown, the HTR8 and BEWO were infected with *shGSDME* lentivirus and the cells were selected by puromycin.

### 4.10 Immunoblots Analysis

The cells were washed once with cold PBS, lysed with cell lysis buffer (Beyotime, Cat. P0013) on ice for 30 min and shake every 10 min, followed by centrifugation at 12 000×g for 30 min. The supernatant was collected and protein concentration were determined using a BCA Protein Assay kit (Thermo, Cat. 23228). Equal amount of protein was separated by sodium dodecylsulfate‐polyacrylamide gradient gel electrophoresis, and transferred onto a PVDF membrane, followed by primary and secondary antibodies incubation. Immunoblots images were developed using the SageCapture gel imaging system (ChampChemi610, Beijing, China). The Image J was employed for quantitative analysis and the relative protein expression level was determined by using ERK2 as loading controls. Primary antibodies: CASP1/p20/p10 Polyclonal Antibody (Proteintech, Cat. 22915‐1‐AP, dilution ratio of 1:1000), Cleaved Gasdermin D (CST, Cat. 36425S, dilution ratio of 1:1000), GSDME‐N‐terminal (Abcam, Cat. ab215191, dilution ratio of 1:1000), HMGB1 antibody (Abcam, Cat. ab18256, dilution ratio of 1:1000), CASP3 antibody (Sigma, Cat. HPA002643, dilution ratio of 1:1000), Cleaved CASP3 antibody (CST, Cat. 9664S, dilution ratio of 1:1000), MLKL antibody (Abcam, Cat. ab194699, dilution ratio of 1:1000), p‐MLKL (Abcam, Cat. ab196436, dilution ratio of 1:1000), p‐RIPK3 (Abcam, Cat. ab209384, dilution ratio of 1:1000), RIPK3 (Santa, Cat. sc‐374639, dilution ratio of 1:200), ERK2 (Santa, Cat. sc‐1647, dilution ratio of 1:400). All uncropped and unprocessed scans of western blots were supplied in Figure S7.

### 4.11 Immunostaining and Immunofluorescence

All the samples from the biopsy were fixed in formalin and embedded in paraffin for section. The 5 µm tissue sections were deparaffinized and dehydrated, subjected to antigen retrieval by autoclaving in 10 mm sodium citrate solution at 120°C for 15 min. For IHC, endogenous peroxidase activity was quenched in 3% H_2_O_2_ in methanol for 10 min, and sections were blocked in 5% BSA in PBS for 1 h. Primary antibodies against GSDME‐N‐terminal (Abcam, Cat. ab215191, 1:200), CASP3 antibody (Sigma, Cat. HPA002643, 1:200), Cleaved CASP3 antibody (CST, Cat. 9664S, 1:200), MLKL antibody (Abcam, Cat. ab194699, 1:200), RIPK3 (Santa, Cat. sc‐374639, 1:200) were added overnight at 4°C, then slides were washed in PBS three times for 10 min, incubated with secondary antibodies for 1 h at room temperature, and washed again. and incubated with the secondary antibody at room temperature for 1 h. The signals were visualized by diaminobenzidine (DAB) staining (ZSGB‐BIO, Cat. ZLI‐9019) at room temperature. IHC slides were counterstained with hematoxylin and scanned using an Olympus microscope (Cat. BX43). For IF, after antigen retrieval, slides were washed in PBS three times for 10 min and then blocked in 5% BSA in PBS for 1 h. Primary antibodies against GSDME‐N‐terminal (Abcam, Cat. ab215191, 1:200), Cleaved CASP3 antibody (CST, Cat. 9664S, 1:200), Cytokeratin 7 (CK7, Abcam, Cat. Ab9021, 1:200), iNOS (Abcam, Cat. Ab3523, 1:200), CD163(Abcam, Cat. ab182422, 1:200), Anti‐Mannose Receptor (CD206, Abcam, Cat. ab64693, 1:200) were incubated at 4°C overnight, and sections were washed in PBS (three times, 10 min each), followed by incubation with Alexa Fluor secondary antibodies and DAPI (1 µg/mL, Sigma, Cat. D9542), as indicated. Washed slides were mounted with Antifade Mountant (Vector, Cat. H1200). Images were taken using a fluorescence microscope (Nikon, Cat. A1R+N‐STORM).

Three randomly selected fields per slide were captured at 200x magnification for analysis. IF signals were quantified as mean fluorescence intensity (MFI) and the percentage of positive cells using standardized thresholding in ImageJ.

### 4.12 Whole Mount Immunofluorescence

Assembloids in culture were directly fixed in 4% paraformaldehyde (PFA) for 30 min. This was followed by a 60 min permeabilization and blocking step using PBS supplemented with 0.2% Triton X‐100 and 5% Bovine Serum Albumin (BSA). For immunofluorescence staining, the assembloids were incubated with primary antibodies overnight at 4°C. After three washes in PBS (10 min each), the assembloids were incubated with Alexa Fluor secondary antibodies and DAPI (1 µg/mL, Sigma, Cat. D9542). The washed assembloids were then mounted with Fluoromount‐G (Southbiotech, Cat. 0100–01). Images were captured using a ZEISS 800 Laser Scanning Confocal Microscope.

### 4.13 Flow Cytometry

For flow cytometry analysis, single‐cell suspensions were obtained by passage through a strainer (70 µm), washed in FACS buffer (PBS with 2% FBS) and stained using the FITC Annexin V Apoptosis Detection Kit Annexin V‐FITC/PI (BD, Cat. 556547) or DAPI (Sigma, Cat. D9542, 0.5 µg/mL) according manufacture's instruction. Flow cytometry was performed on a Thermo Fisher Attune NxT flow cytometer and data were processed with FlowJo software.

### 4.14 Cell Cytotoxicity and Cell Viability Assay

The supernatant of trophoblast cells of indicated lines was collected and LDH release was quantified using CytoTox96 Non‐Radioactive Cytotoxicity Assay (Promega, Cat. G1780) according to the manufacturer's instructions. Percentage cytotoxicity was calculated based on maximum LDH release from unstimulated cells lysed with Lysis Solution. Cell viability under 24 h treatment was determined via the Cell Counting Kit‐8 (CCK‐8, DOJINDO, Cat. CK04).

### 4.15 Detection of Inflammatory Cytokines

Detection of inflammatory cytokines was performed using the LEGENDplex Multi‐Analyte Flow Assay Kit (Human Inflammation Panel 1 (13‐plex), Biolegend, Cat. 740808) following the manufacturer's instructions. In brief, supernatants of HTR8 were collected after treatment for 24 h, then diluted 1:1 with assay buffer. 25 µL mixed beads were added to each well and incubated for 2 h at room temperature with 1000 rpm shaking. The wells were washed once with 1xWash Buffer, followed by detection antibodies incubation for 1 h, the 25 µL SA‐PE was added to each well directly and incubated for 30 min. The beads were washed once and resuspended with 300 µL of 1xWash Buffer. The data were collected on a Attune NxT Flow cytometer (Invitrogen).

### 4.16 ELISA Analysis

To measure IL‐1β and IL‐18 released in vitro, HTR8, TSC or Bewo cells were stimulated with T/S for indicated hours, and the supernatants were collected and detected using Duoset ELISA assay kits for human IL‐1β (Proteintech, Cat. KE00021) and IL‐18 (abcam, Cat. ab215539) according to manufacturer's protocols. To measure mouse serum IL‐1β and IL‐18 released in vivo, pregnant mice treated with 0.9% Nacl or T/S were sacrificed, and whole blood was collected at E11.5, followed by centrifuge at 2000 g, the serum (50 µL) was extracted and detected with mouse IL‐1β ELISA Kit (Cusabio, Cat. CSB‐E08054m) and mouse IL‐18 ELISA Kit (eBiosciences, Cat. E‐EL‐M0730c) following by the manufacturer's instructions. To measure human serum IL‐1β, IL‐18 and CASP3, the serum (50 µL) was extracted and detected with human human IL‐1β (Proteintech, Cat. KE00021), IL‐18 (abcam, Cat. ab215539), BAX (GILED BIOTECHNOLOGY, Cat. J20366) and CASP3 (GILED BIOTECHNOLOGY, Cat. J21110) following by the manufacturer's instructions.

### 4.17 Statistics and Reproducibility

Statistical analysis was performed with GraphPad Prism (v9). Statistical analyses were performed using two‐tailed unpaired Student's *t*‐test and among more than two groups by one‐way analysis of variance. Each experiment was performed in three times independently, otherwise indicated. Data were shown as the mean ± SEM, ns, not significant, ^*^ *p* < 0.05, ^**^ *p* < 0.01, and ^***^ *p* < 0.001, ^****^ *p* < 0.001.

## Author Contributions

B.H., W.D., S.B. and Z.F. conducted the experiments; L.H. and L.Z. collected human samples; Y.W., W.S., T.L., L.D., Z.T. and J.K. analyzed the proteomics data. H.W., J. C., D.C. and S.Z. designed experiments and wrote the manuscript.

## Conflicts of Interest

The authors declare no conflicts of interest.

## Supporting information




**Supporting File 1**: advs73918‐sup‐0001‐SuppMat.docx

## Data Availability

The data that support the findings of this study are available on request from the corresponding author. The data are not publicly available due to privacy or ethical restrictions.
