# Supplementary material for: Caspase‐3/GSDME‐Mediated Trophoblast Pyroptosis and Reciprocal Macrophage Polarization Contribute to Inflammation in Early‐Onset Preeclampsia
Source: Adv Sci (Weinh). 2026 Jan 20;13(18):e16948. doi: 10.1002/advs.202516948 (PMC13042631; doi:10.1002/advs.202516948)
Supplement: Supplementary file 1 — Supporting File 1: advs73918‐sup‐0001‐SuppMat.docx [file ADVS-13-e16948-s001.docx]

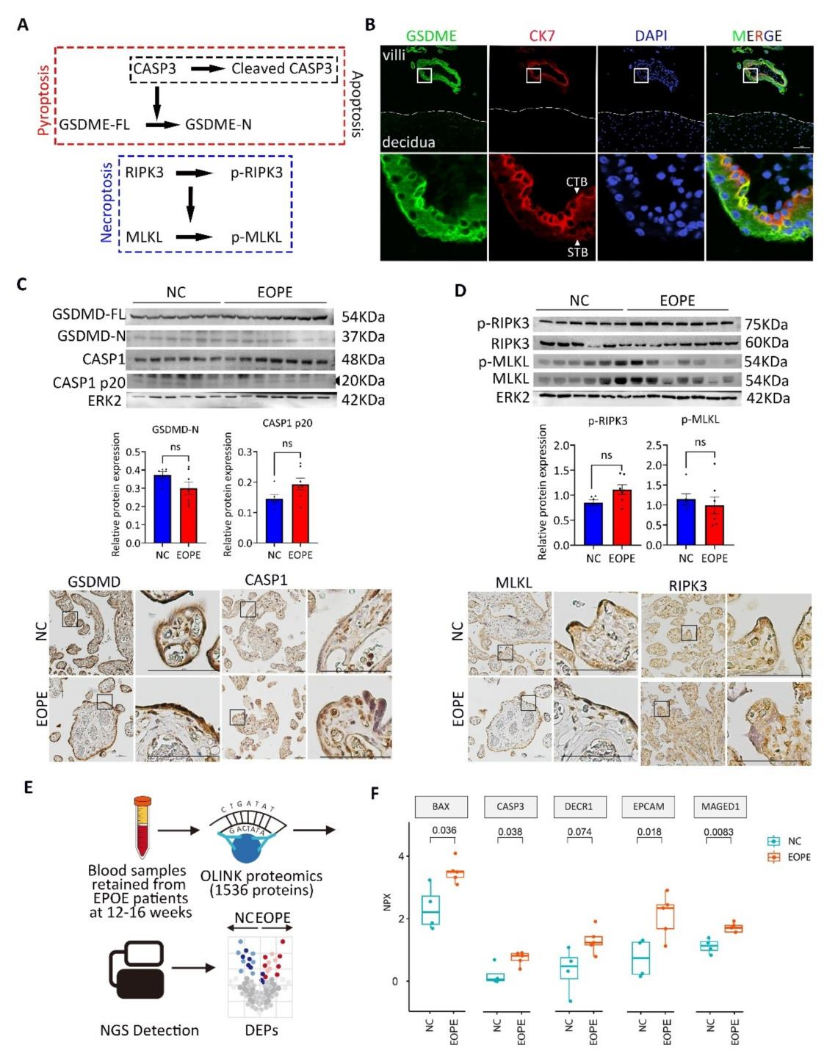
 **Supplementary Figure 1. Detection of cell death pathway-related proteins in placental tissues and plasma from EOPE.**

(A) Schematic diagram of apoptosis, necroptosis and pyroptosis pathways. (B) Representative Immunofluorescence pictures of GSDME, CK7 and DAPI in the normal pregnant placenta villi and decidua at the first trimester. Scale bars, 100 μm. (C) Immunoblots of pyroptosis-related proteins (GSDMD-FL, GSDMD-N, CASP1 and CASP1 p20) in placental villous lysates from controls (n=6) and EOPE (n=7). ERK2 was used as a loading control. Representative immunohistochemical staining of GSDMD and CASP1 in placental villi from NC (n=6) and EOPE (n=7) groups. Scale bars: 50 μm. (D)Immunoblots of necroptosis-related proteins (p-RIPK3, RIPK3, MLKL and p-MLKL) in placental villous lysates from controls(n=6) and EOPE(n=7). ERK2 was used as a loading control. Representative immunohistochemical staining of MLKL and RIPK3 in placental villi from NC (n=6) and EOPE (n=6) groups. Scale bars: 50 μm. (E) Schematic diagram of proteomics using Olink sequencing platform. (F) Box plot showing the expression levels of the five most upregulated proteins among the top 20 differentially expressed proteins in blood from EOPE compared to controls. NC, control; EOPE, Early-onset preeclampsia.


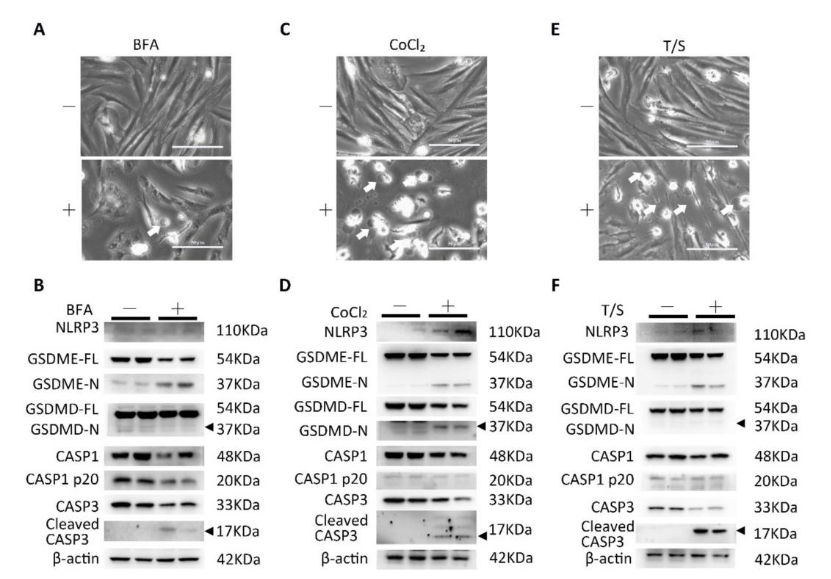


**Supplementary Figure 2. GSDME‐mediate pyroptosis was induced upon activation of apoptosis in primary human trophoblasts.**

(A) Phase-contrast images of primary human trophoblasts (PHTs) treated with brefeldin A (BFA, ER stress inducers), 1 μg/ml, after 24 hours. White arrows indicated the swelling cells. Scale bars, 50 μm. (B) Immunoblots of NLRP3, GSDME-FL, GSDME-N, GSDMD-FL, GSDMD-N, CASP1, CASP1 p20, CASP3 and cleaved CASP3 in PHTs treated with BFA after 24 hours. β-actin was used as a loading control. (C) Phase-contrast images of PHTs treated with CoCl_2_ after 24 hours. White arrows indicated the swelling cells. Scale bars, 50 μm. (D) Immunoblots of NLRP3, GSDME-FL, GSDME-N, GSDMD-FL, GSDMD-N, CASP1, CASP1 p20, CASP3 and Cleaved CASP3 in PHTs treated with CoCl_2_ after 24 hours. β-actin was used as a loading control. (E) Phase-contrast images of PHTs treated with T/Safter 24 hours. White arrows indicated the swelling cells. Scale bars, 50 μm. (F) Immunoblots of NLRP3, GSDME-FL, GSDME-N, GSDMD-FL, GSDMD-N, CASP1, CASP1 p20, CASP3 and Cleaved CASP3 in PHTs treated with T/S after 24 hours. β-actin was used as a loading control. T/S, SM164 and TNFα.


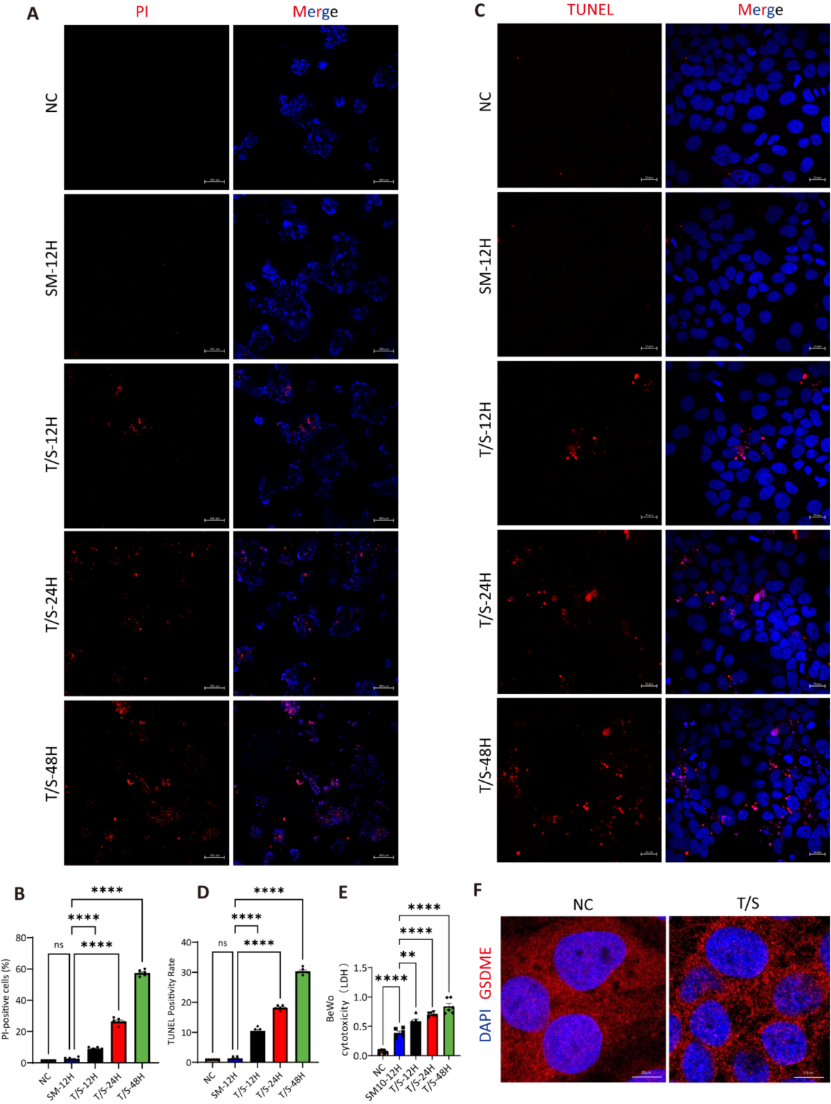


**Supplementary Figure 3. GSDME‐mediate pyroptosis was induced upon activation of apoptosis.**

(A) Representative bright field and fluorescent images of BeWo cells stained with propidium iodide (PI) under the indicated treatments: NC (control), SM‑12h (SM164 for 12 h), T/S‑12h, T/S‑24h, T/S‑48h (TNFα + SM164 for 12, 24 or 48 h). Scale bars, 100 μm. (B) Quantification of PI‑positive cells after T/S treatment. Data are mean ± SEM from n = 3 independent experiments; each experiment included duplicate wells and three randomly selected fields were counted per well. Statistical analysis by one‑way ANOVA; ****p < 0.0001. (C) Representative TUNEL images showing time‑dependent increase in DNA fragmentation in BeWo cells treated with T/S (same treatment groups as in A). (D) Quantification of TUNEL‑positive cells after T/S treatment. Data are mean ± SEM, n = 3 independent experiments; duplicates per experiment, three fields counted per sample. One‑way ANOVA; ****p < 0.0001. (E) LDH release from BeWo cells following T/S treatment at 12, 24 and 48 h. Data are mean ± SEM, n = 3 independent experiments; duplicates per experiment. One‑way ANOVA; ****p < 0.0001, **p < 0.01. (F) Immunofluorescence localization of GSDME in BeWo cells treated without or with T/S for 24 h. Prior to T/S treatment GSDME staining is diffuse throughout the cytoplasm; after 24 h T/S treatment GSDME redistributes to membrane‑associated puncta consistent with formation of GSDME‑N membrane pores. Scale bars = 10 μm. NC, control; T/S, SM164 and TNFα.


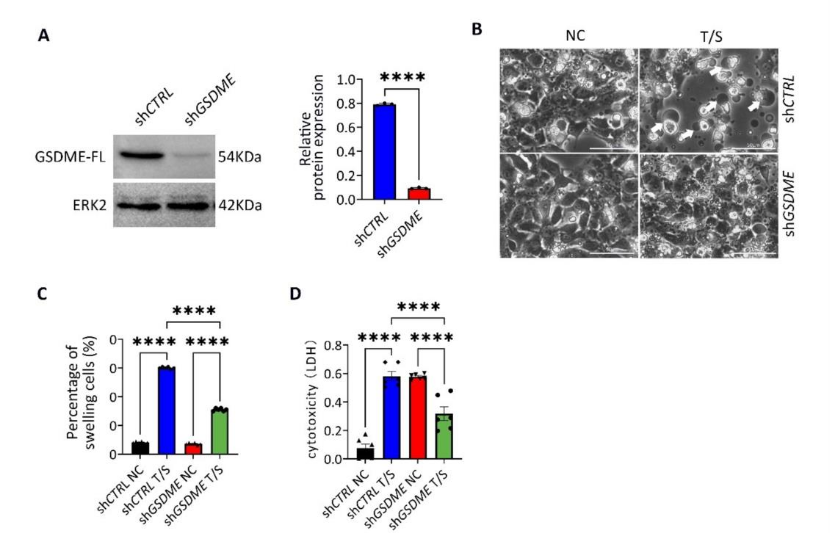
 **Supplementary Figure 4. Inhibition of GSDME reduces pyroptosis-like phenotype.**

(A) Immunoblots of GSDME in sh*CTRL* and sh*GSDME* BeWo cells. ****p<0.0001. (B-C) Phase-contrast images of BeWo cells (sh*CTRL* and *shGSDME*) treated with T/S after 24 hours. Arrows, the swelling cells. Scale bars, 50 μm. (C) Percentages of swelling cells after T/S treatment. Error bars, mean ± SEM, n =3, duplicate each time, three representative fields per group were analyzed. The data were analyzed with a one-way ANOVA. **** p<0.0001. ns, not significant. (D) Comparison of LDH release-based cell death of BeWo cells (sh*CTRL* and sh*GSDME*) treated with T/S after 24 hours. Error bars, mean ± SEM, n =3, duplicate each time. The data were analyzed with a one-way ANOVA, **** p<0.0001, ns, not significant. NC, control; T/S, SM164 and TNFα.


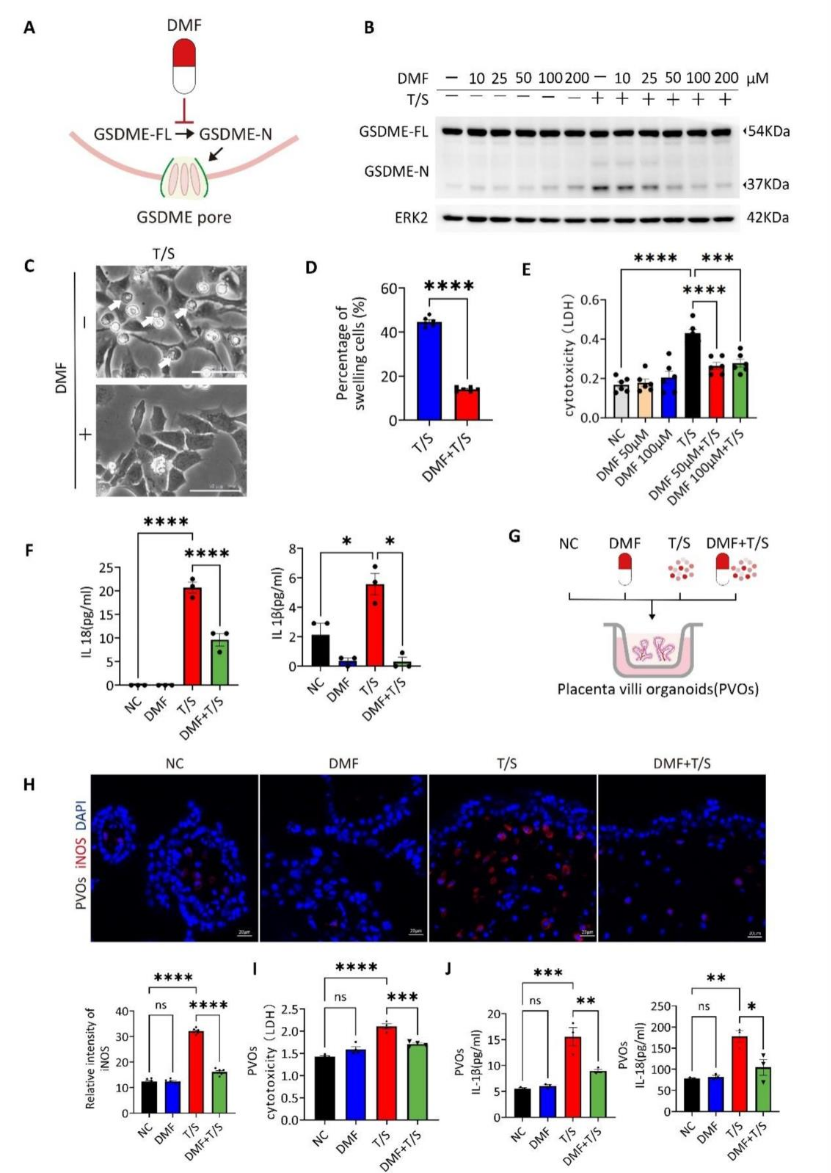


**Supplementary Figure 5. Targeting GSDME cleavage by DMF reduced pyroptosis in human trophoblast cells and inflammatory macrophages in placenta villi organoid.**

(A)Scheme of the mechanism of DMF on inhibiting GSDME cleavage. (B)Immunoblots of GSDME-FL and GSDME-N in HTR8/SVneo cells, as well as HMGB1 in the supernatant, in HTR8/SVneo cells treated with T/S and DMF. ERK2 was used as a loading control. (C) Phase-contrast images of HTR8/SVneo cells treated with T/S and DMF after 24 hours. Arrows indicated the swelling cells. Scale bars, 50 μm. (D) Percentages of swelling cells in T/S and T/S plus DMF treatment after 24h. n =3, duplicate each time, three representative fields per group were analyzed. Data represent mean ± SEM, **** p<0.0001. (E) Comparison of LDH release-based cell death of HTR8/SVneo cells treated with T/S and DMF (0, 50, 100μM) after 24 hours. n =3, duplicate each time. Data represent mean ± SEM, **** p<0.0001, *** p<0.001, ns, not significant. (F) Relative levels of, IL-18, IL-1β in the supernatant of HTR8/SVneo cells treated with T/S and DMF using the ELISA kits. All data were representative of three independent experiments. * p<0.05, **** p<0.0001. (G) Schematic diagram of placenta villi organoid treated with DMF and T/S. (H) Representative Immunofluorescence staining images of iNOS and DAPI of PVOs treated DMF and T/S after 24 hours. The quantification of relative level of iNOS in the PVOs treated DMF and T/S after 24 hours. Error bars, mean ± SEM. The data were analyzed with a one-way ANOVA, n =3, duplicate each time, three representative fields per slide were analyzed, **** p<0.0001. Scale bars, 20 μm. (I)Comparison of LDH release-based cell death of placenta villi organoid treated with DMF and T/S after 24 hours. Error bars, mean ± SEM, n ≥3. The data were analyzed with a one-way ANOVA, **** p<0.0001, *** p<0.001. (J) IL-18 and IL-1β level in the supernatant of PVOs treated DMF and T/S after 24 hours using the ELISA kit. Error bars, mean ± SEM. The data were analyzed with a one-way ANOVA, n ≥ 3, *** p<0.001, ** p<0.01, * p<0.05, ns, not significant. NC, control; T/S, TNFα and SM164; DMF, dimethyl fumarate.


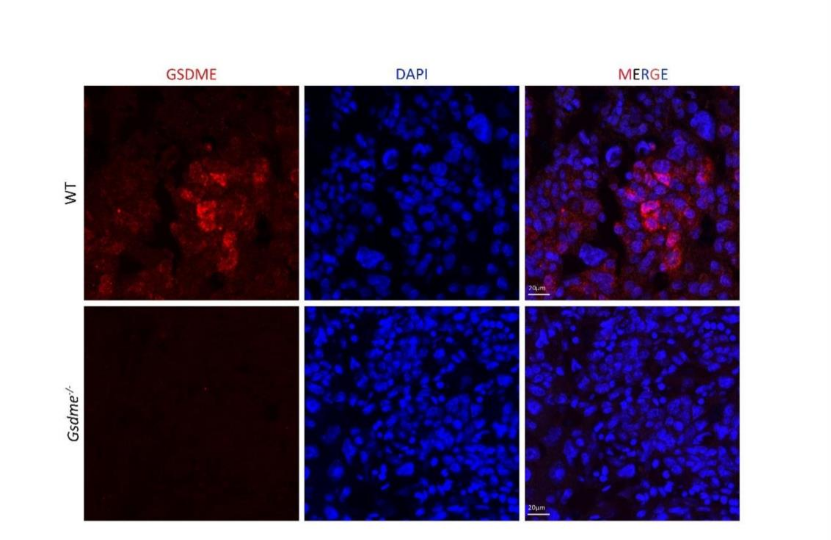
**Supplementary Figure 6.** Representative Immunofluorescence staining images of GSDME in placenta tissue of wild type and *Gsdme^-/-^* mice. Scale bars, 20 μm.


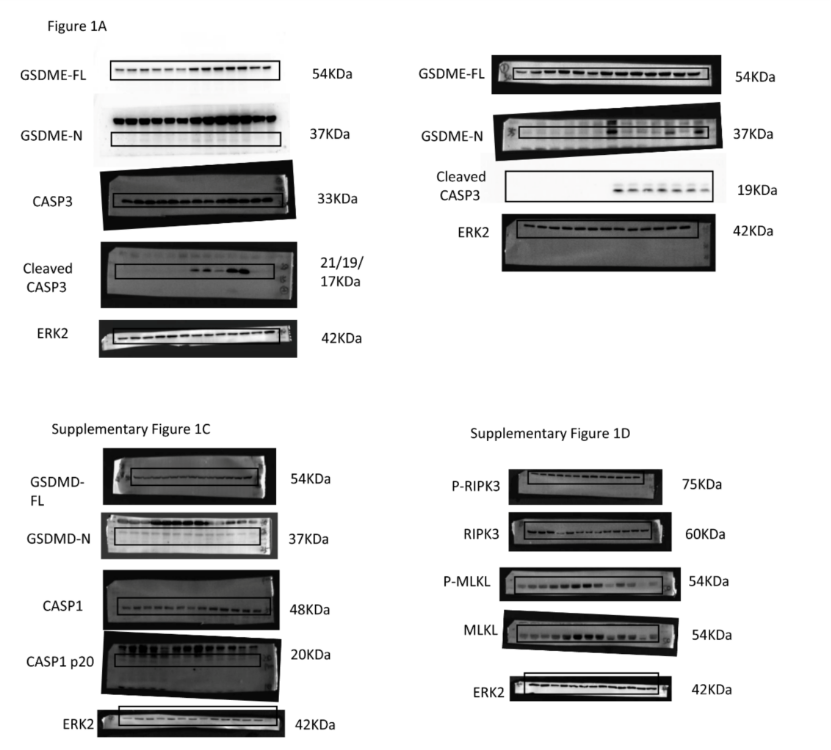


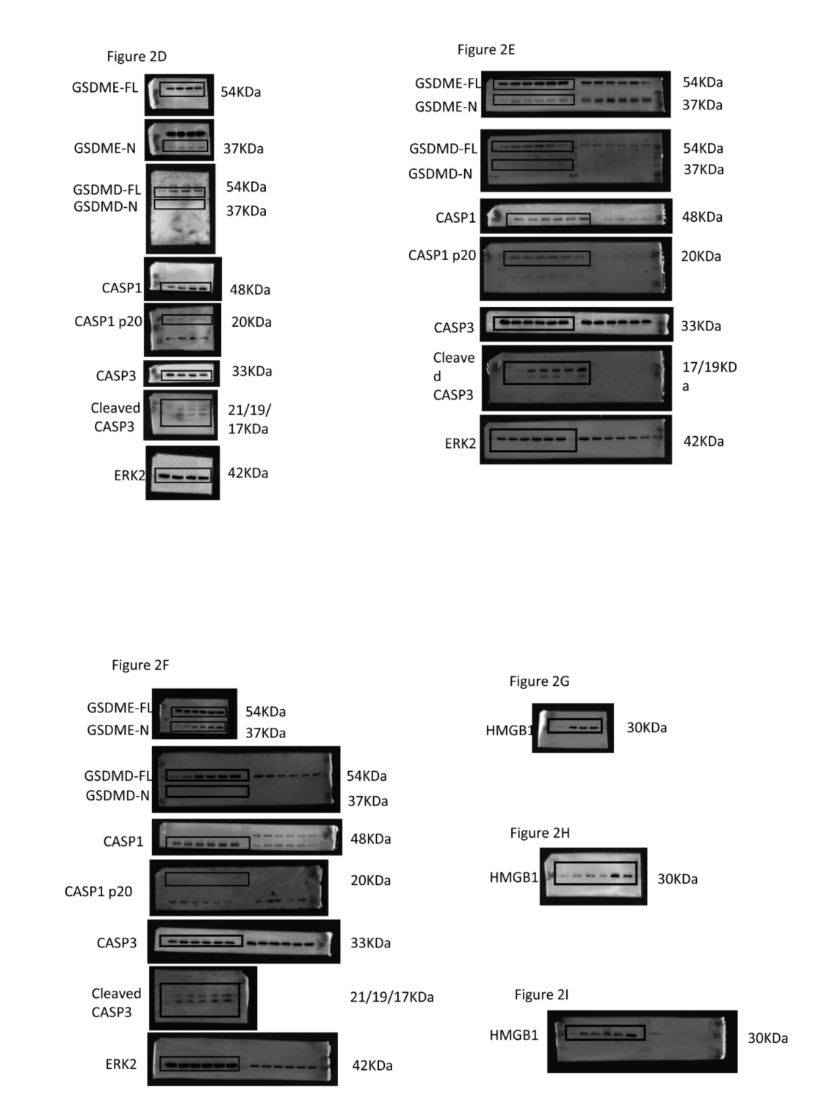


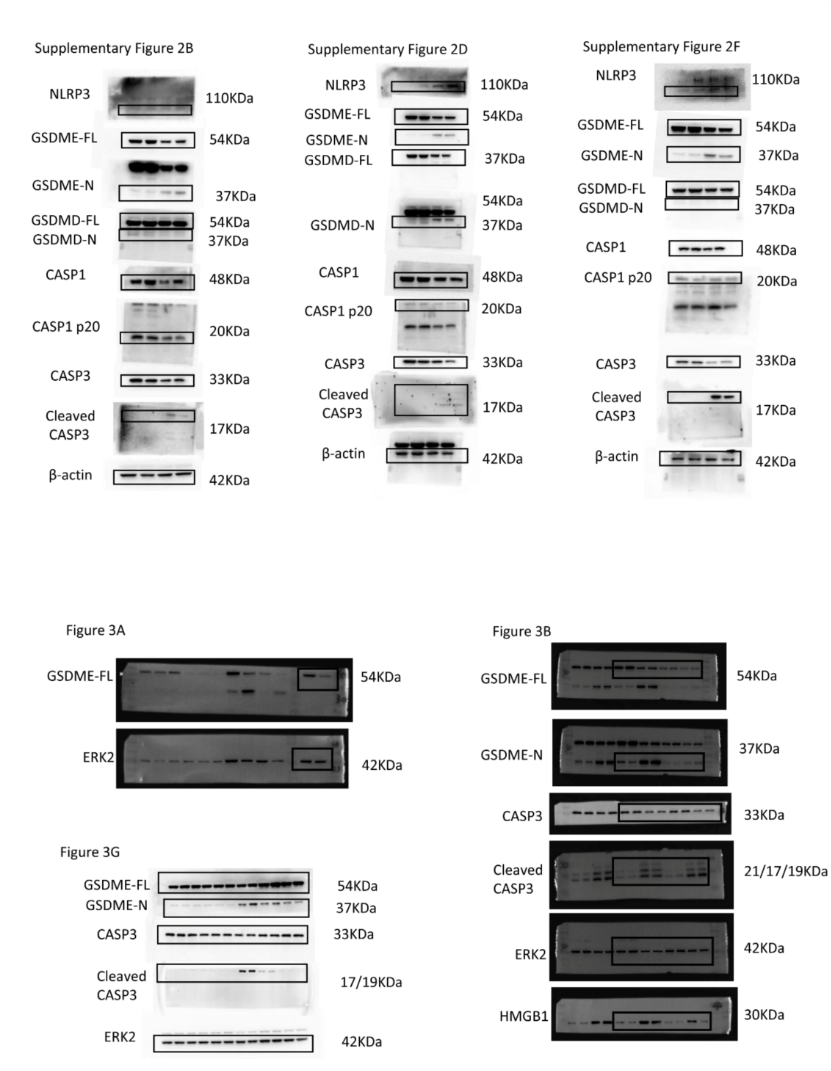


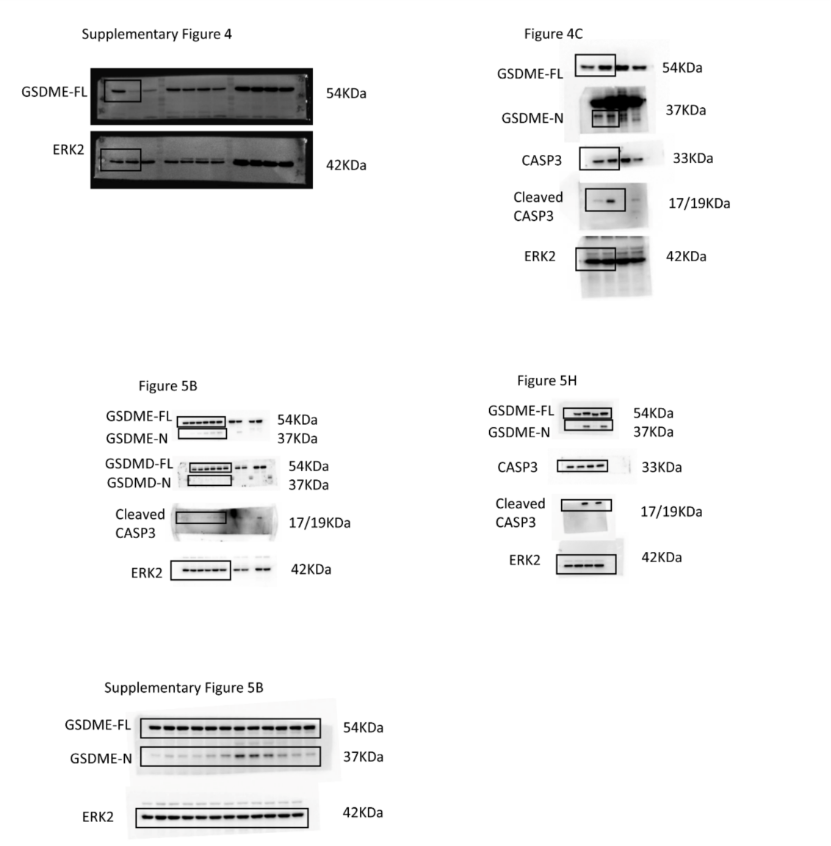


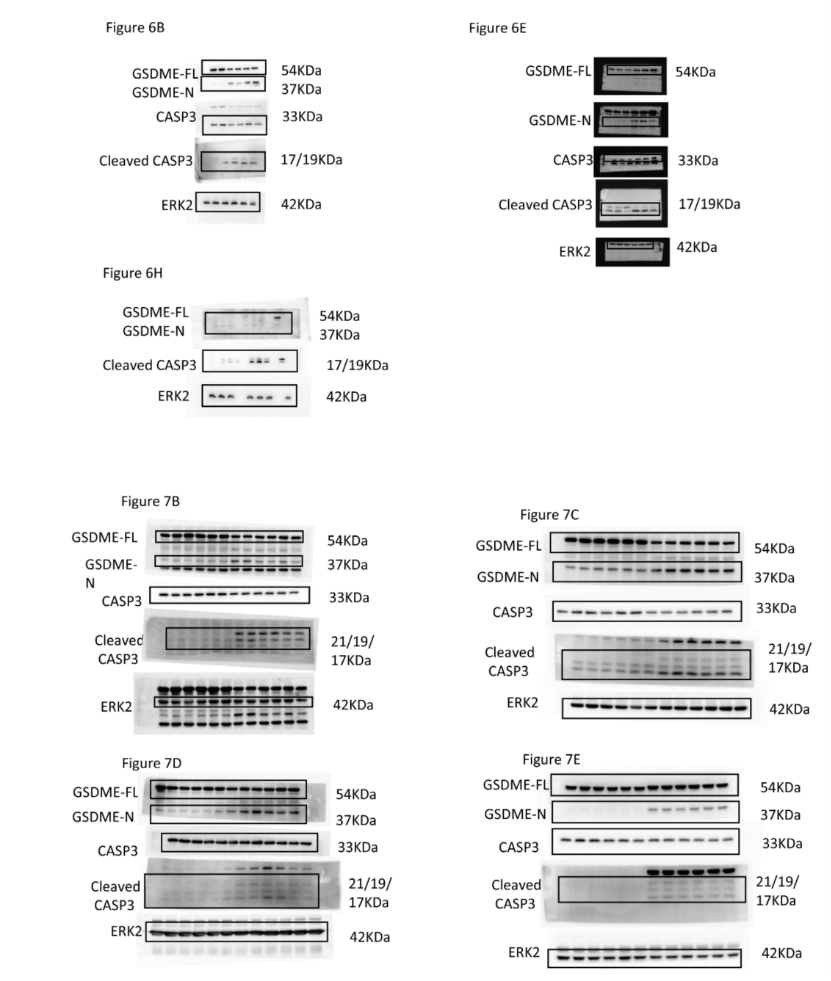


**Supplementary Figure 7. Unprocessed images of immunoblotting.**

PVDF membranes were cut into several small pieces to incubate with different antibodies for immunoblotting. Black boxes indicate images showed in relevant figures.

**Supplementary Table 1.** **Clinical characteristics of early-onset preeclampsia (EOPE) patients and matched controls**

Note： Sample analyzed by Western blotting in Figure 1A.

| group | Maternal age(years) | | Gestational age at  delivery(weeks) | BMI(kg/m^2^) | | | Maxmum SBP(mmHg) | Maxmum DBP(mmHg) | Fetus gender | Proteinuria |
| --- | --- | --- | --- | --- | --- | --- | --- | --- | --- | --- |
| EOPE | 31 | 29 | | | 27.3 | 160 | | 85 | male | + |
| EOPE | 31 | 31 | | | 30.2 | 140 | | 84 | male | + |
| EOPE | 34 | 32 | | | 20.8 | 158 | | 97 | female | + |
| EOPE | 32 | 31 | | | 22.3 | 139 | | 98 | female | + |
| EOPE | 34 | 29 | | | 25.3 | 138 | | 94 | male | + |
| EOPE | 29 | 30 | | | 31.37 | 158 | | 95 | female | - |
| EOPE | 30 | 29 | | | 27.3 | 160 | | 84 | female | + |
| EOPE | 31 | 36.6 | | | 22.3 | 150 | | 84 | male | + |
| EOPE | 30 | 29.9 | | | 24.2 | 168 | | 83 | male | + |
| EOPE | 32 | 31.6 | | | 23.8 | 153 | | 89 | female | + |
| EOPE | 30 | 30.9 | | | 23.53 | 139 | | 89 | male | - |
| EOPE | 32 | 27.9 | | | 24.32 | 142 | | 91 | female | + |
| EOPE | 31 | 30.9 | | | 27.37 | 160 | | 95 | male | + |
| EOPE | 32 | 30.2 | | | 27.3 | 152 | | 84 | female | + |
| NC | 27 | 31 | | | 25.2 | 118 | | 75 | male | - |
| NC | 31 | 29 | | | 23.5 | 124 | | 70 | female | - |
| NC | 30 | 33 | | | 24.3 | 129 | | 71 | male | - |
| NC | 32 | 28 | | | 24.5 | 109 | | 78 | female | - |
| NC | 28 | 32 | | | 22.2 | 127 | | 70 | male | - |
| NC | 29 | 31 | | | 22.8 | 119 | | 69 | male | - |
| NC | 36 | 30.4 | | | 26 | 121 | | 63 | female | - |
| NC | 35 | 31.7 | | | 24.83 | 130 | | 70 | female | - |
| NC | 28 | 31.3 | | | 23.9 | 107 | | 71 | male | - |
| NC | 33 | 31.9 | | | 23.5 | 121 | | 68 | female | - |
| NC | 30 | 30.7 | | | 26 | 116 | | 73 | female | - |
| NC | 24 | 30 | | | 23.83 | 120 | | 66 | male | - |

In the control group, preterm delivery was caused by factors such as cervical insufficiency, iatrogenic factors, and preterm premature rupture of membranes.

**Supplementary Table 2. Clinical characteristics of study participants**

Serum samples for ELISA detection in Figure 1G-H

|  | PE group | | | | | control group | P |
| --- | --- | --- | --- | --- | --- | --- | --- |
| sample size | | | | | N=12 | N=12 |  |
| Maternal age(years) | | 30±0.9045 | | | | 30.58±1.282 | 0.7136 |
| Gestational age at delivery(weeks) | 31.06（30.04, 32.08） | | | | | 33.31(32.38, 34.24) | ＜.01** |
| BMI(kg/m2) | 25.7±0.3764 | | | | | 24.26±0.43 | ＜0.05* |
| primipara, n(%) | 5(41.7) | | | | | 6(50) |  |
| Maximum SBP(mmHg) | | | | 151.1±5.332 | | 113.8±1.886 | ＜0.001*** |
| MaximumDBP(mmHg) | 95.92±3.545 | | | | | 73.42±2.047 | ＜0.001*** |
| Neonatal birth weight(g) | | | 2206±106.3 | | | 3013±67.74 | ＜0.0001**** |
| Sample collection time(weeks) | 24.58±0.14868 | | | | | 24.5±0.1508 | 0.6977 |
| Proteinuria, n(%) | 9(75) | | | | | 0 |  |

In the control group, preterm delivery was caused by factors such as cervical insufficiency, iatrogenic factors, and preterm premature rupture of membranes.

Samples for Western blotting in Supplementary Figure 1B-1C

|  | PE group | | control group | P |
| --- | --- | --- | --- | --- |
| sample size | N=7 | | N=6 |  |
| Maternal age(years) | 31.57±0.7190 | | 29.5±0.7638 | 0.0743 |
| Gestational age at delivery(weeks) | 30(29,31) | | 31(28.75,32.25) | 0.5541 |
| primipara, n(%) | 3(42.8) | | 2(33.3) |  |
| Maximum SBP(mmHg) | | 150.4±4.058 | 121±2.978 | ＜0.001*** |
| Maximum DBP(mmHg) | | 91± | 72.17± | ＜0.0001**** |
| Neonatal birth weight(g) | 2100(1540,2500) | | 2700(2388,2925) | 0.0261* |
| Proteinuria | 6(85.6) | | 0 |  |

In the control group, preterm delivery was caused by factors such as cervical insufficiency, iatrogenic factors, and preterm premature rupture of membranes.

**Supplementary Table 3. Clinical Features of patients for OLINK proteomics**

| group | | Sample number | Maternal age(years) | | Gestational age at delivery(weeks) | | BMI(kg/m^2^) | Primipara | Maximum SBP(mmHg) | Maximum DBP(mmHg) | Neonatal birth weight(g) | Sample collection time(weeks) | Proteinuria |
| --- | --- | --- | --- | --- | --- | --- | --- | --- | --- | --- | --- | --- | --- |
| PE | 19B6909223 | | | 25 | 36.6 | 28.34 | | Yes | 150 | 79 | 3040 | 12.9 | - |
| PE | 19B6909252 | | | 37 | 34.7 | 31.2 | | Yes | 134 | 84 | 2000 | 14 | + |
| PE | 19B3757678 | | | 33 | 31.6 | 18.8 | | no | 153 | 96 | 1280 | 12.3 | + |
| PE | 19B2953258 | | | 36 | 33.9 | 23.53 | | no | 128 | 89 | 1670 | 13.9 | + |
| PE | 19B2084329 | | | 34 | 27.9 | 26.3 | | Yes | 130 | 90 | 670 | 16.4 | + |
| NC | 19B3983464 | | | 36 | 37.1 | 22.65 | | no | 120 | 75 | 3060 | 12.4 | - |
| NC | 19B3983179 | | | 35 | 38.4 | 21.83 | | no | 132 | 79 | 2790 | 18 | - |
| NC | 19B3982793 | | | 28 | 39.3 | 22.5 | | no | 107 | 71 | 2710 | 12.3 | - |
| NC | 19B2084520 | | | 36 | 39.7 | 21.2 | | no | 122 | 75 | 3260 | 13.6 | - |
| NC | 77381 | | | 33 | 39.4 | 22.3 | | no | 118 | 73 | 3160 | 13.4 | - |
| NC | 4025034943 | | | 23.83 | 39 | 23.83 | | no | 118 | 75 | 3630 | 12.4 | - |

In the control group, preterm delivery was caused by factors such as cervical insufficiency, iatrogenic factors, and preterm premature rupture of membrane.
